# Regulation of the lncRNA *malat1*/Egr1 axis by Wnt, Notch, and TGF-β signaling: a key mechanism in retina regeneration

**DOI:** 10.1093/narmme/ugaf024

**Published:** 2025-07-21

**Authors:** Sharanya Premraj, Poonam Sharma, Mansi Chaudhary, Pooja Shukla, Kshitiz Yadav, Omkar Mahadeo Desai, Rajesh Ramachandran

**Affiliations:** Department of Biological Sciences, Indian Institute of Science Education and Research, Mohali, Knowledge City, SAS Nagar, Sector 81, Manauli PO, Mohali, 140306 Punjab, India; Department of Biological Sciences, Indian Institute of Science Education and Research, Mohali, Knowledge City, SAS Nagar, Sector 81, Manauli PO, Mohali, 140306 Punjab, India; Department of Biological Sciences, Indian Institute of Science Education and Research, Mohali, Knowledge City, SAS Nagar, Sector 81, Manauli PO, Mohali, 140306 Punjab, India; Department of Biological Sciences, Indian Institute of Science Education and Research, Mohali, Knowledge City, SAS Nagar, Sector 81, Manauli PO, Mohali, 140306 Punjab, India; Department of Biological Sciences, Indian Institute of Science Education and Research, Mohali, Knowledge City, SAS Nagar, Sector 81, Manauli PO, Mohali, 140306 Punjab, India; Department of Biological Sciences, Indian Institute of Science Education and Research, Mohali, Knowledge City, SAS Nagar, Sector 81, Manauli PO, Mohali, 140306 Punjab, India; Model Systems for Infection and Immunity (MSYS) Helmholtz Centre for Infection Research, Inhoffenstr. 7, Braunschweig, 38124, Germany; Department of Biological Sciences, Indian Institute of Science Education and Research, Mohali, Knowledge City, SAS Nagar, Sector 81, Manauli PO, Mohali, 140306 Punjab, India

## Abstract

Unlike mammals, upon injury, adult zebrafish retinas rely on their ability to reprogram resident Müller glia to progenitor cells, enabling regeneration and restoration of vision. Retina regeneration remains incomplete in mammals despite extensive efforts to stimulate zebrafish regenerative conditions. Here, we show that the zebrafish lncRNA *malat1 (metastasis-associated lung adenocarcinoma transcript 1*), crucial for many biological functions, plays essential roles during retina regeneration. We demonstrate that zebrafish *malat1* functions through an Egr1-dependent axis, modulated by Wnt, Notch, and TGF-β signaling pathways, and is necessary for effective retina regeneration. We performed RNA immunoprecipitation to establish the association of zebrafish *malat1* with epigenetic modifiers Ezh2 and Hdac1 in the regenerating retina. Moreover, we uncover that the antisense lncRNA *talam1*, which regulates *malat1* availability, is differentially regulated in zebrafish and mice, highlighting species-specific gene regulatory mechanisms after retinal injury. Cells with active TGF-β signaling stabilize mouse *Malat1* while the same signaling destabilizes zebrafish *malat1*. Our findings uncover a previously unknown role of *malat1*/Egr1 axis as a crucial regulator of retina regeneration, shedding light on its possible influence on the different regenerative abilities of vertebrates.

## Graphical Abstract

**Graphical Abstract:**
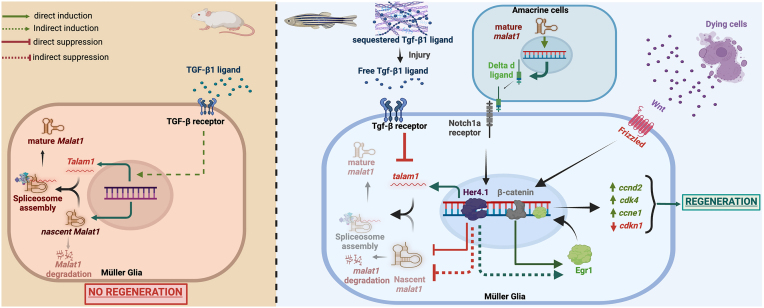

## Introduction

Müller glia-dependent retina regeneration has been extensively studied in zebrafish and mouse models [1]. Unlike mammals, vertebrates such as fish and frogs have remarkable abilities to regenerate the retina [2]. Retina regeneration adopts several gene regulatory networks associated with embryonic development, cancer, and wound healing [3, 4]. Several regeneration-associated pathways that activate different gene regulatory networks essential to zebrafish retina regeneration are either suboptimal or antagonistic in the injured mice retina. Forced emulated zebrafish-specific retinal gene expression in mice often resulted in better regenerative response, suggesting the relevance of these regeneration-associated genes (RAGs) [5].

Although many noncoding RNAs are involved in several biological phenomena [6, 7], this study mainly focuses on the long noncoding RNA *malat1* (*metastasis-associated lung adenocarcinoma transcript 1*). It has been extensively demonstrated to be relevant to many biological functions, including embryonic development [8], cellular proliferation [9], tissue differentiation [10], homeostasis [11], and cancer [12]. It also plays important roles in wound healing [13, 14], cancer initiation, and metastasis [15–17]. *Malat1* is known to promote cellular proliferation and is considered to be a curative target for cancer [18, 19] and metabolic syndromes such as Type 2 diabetes [20]. The roles of *malat1* are explored and demonstrated in bone fracture healing [21], peripheral nervous system repair [22], and muscle [23] and endothelium [24] regeneration. Hence, we anticipated the contribution of *malat1* to retina regeneration, a context in which its function remains unexplored. Interestingly, *malat1* knock-out mice show no overt phenotype under normal conditions [25], suggesting possible compensatory mechanisms or context-dependent functions, further highlighting the use of a regeneration-competent model like zebrafish to understand the potential role of *malat1* in tissue repair.

Here, we investigated the regulation of *malat1* during zebrafish retina regeneration and its broader role in coordinating gene expression programs essential for mounting a robust regenerative response. During retina regeneration, we explored the link between zebrafish *malat1* and different signaling pathways, such as Tgf-β (Transforming growth factor- beta), Wnt, and Delta-Notch signaling. Furthermore, we demonstrated that zebrafish and mice retinas exhibit distinct responses to injury, potentially driven by the Tgf-β signaling-mediated regulation of *malat1* expression in the injured retina of both species.

## Materials and methods

### Zebrafish husbandry, retinal injury, and pharmacological agents

In circulating aquatic systems, wild-type zebrafish (*Danio rerio*) were maintained at 26–28.5°C on a 14:10-h light/dark cycle. Embryos were obtained by natural breeding. Transgenic *1016 tuba1a: GFP* zebrafish (Fausett & Goldman, 2006) were also used in this study. Retinal injuries were induced in 8–12-month-old fish under tricaine methane sulfonate anesthesia with a 30G needle (Fausett & Goldman, 2006; Sharma & Ramachandran, 2019).

C57BL/6 mice were housed on a 12-h light/dark cycle with *ad libitum* food and water. The retinal injury was induced, under isoflurane anesthesia, by injecting 100 mM *N*-methyl d-aspartic acid (NMDA) intravitreally. All procedures followed institutional ethical guidelines and were IAEC approved.

Unless otherwise specified, drug and protein injections into the vitreous were performed during the injury process, using a 5 μl Hamilton syringe, equipped with a 30G needle. All pharmacological agents used namely SB431542, TGF-β inhibitor (Sigma–Aldrich, S4317), XAV939, Wnt signaling inhibitor (Tocris), and glycogen synthase kinase-3β (GSK-3β) inhibitor I (SB216763, EMD Calbiochem), were dissolved in DMSO (Dimethyl sulfoxide), to make stock concentration as 1 mM, which was diluted further in PBS (Phosphate buffered saline) or autoclaved de-ionized water. TGF-β1 protein (Abcam, ab50036) was prepared at 4 mg/ml in 10 mM citric acid, with working concentrations diluted in PBS or autoclaved deionized water. Chemical-mediated injury with NMDA (Sigma, M3262) was performed by delivering it to vitreous humor in zebrafish and mice eyes, through the cornea.

### Transgenic line generation

Transgenic lines were generated using the Tol2 system with *pT2AL200R150G* vector [26]. Approximately 4 kb *malat1* and *talam1* promoters were cloned upstream of GFP in the vector and the purified construct was injected with 25 ng *tol2* mRNA into one-cell zebrafish embryos, then screened for GFP.

### Antisense morpholino transfection and*in vivo* mRNA transfection

For gene knockdown experiments, ∼0.5 μl of Lissamine-tagged morpholinos (MOs) (Gene Tools) at a concentration of 0.125–1.0 mM were administered during retinal injury using a 5 μl Hamilton syringe. To facilitate MO delivery into retinal cells, electroporation was performed following the method described by Fausett *et al.* [27]. Sequences of MOs used in the study are:

Control MO: 5′CCTCTTACCTCAGTTACAATTTATA-3′ [27]

*ascl1a* MO: 5′ATCTTGGCGGTGATGTCCATTTCGC-3′ [27]

*malat1* MO: 5′CCACCAGGGTCTTTTGCTTTTTTTC-3′ [8]

*egr1* MO: 5′-GCA GCC ATC TCT CTG GAG TGT GCT C-3′ [28]

*her4.1* MO: 5′-TTGATCCAGTGATTGTAGGAGTCAT-3′ [29]

For *in vivo* mRNA transfection, the pCS2 vector harboring the full-length coding sequence of genes, in correct orientation was linearized using NotI/KpnI, and capped mRNA (messenger RNA) was synthesized *in vitro* using mMESSAGE mMACHINE Kit (Ambion), as per manufacturer’s instructions. A mix containing lipofectamine, HBSS (Hanks' balanced salt solution), and mRNA was prepared as described in [29–31]. The mix was delivered intravitreally through the cornea using a Hamilton syringe. The transfection mixture containing *gfp* mRNA was parallelly injected into the control retina. For embryos, ∼200 nl of MO or mRNA mix was injected in one cell stage.

### Primers and plasmid construction

Supplementary Table S1 lists all the primers used in the study for quantitative real-time PCR (qPCR), cloning, SDM (site-directed mutagenesis), and probe template. The coding sequence of *malat1* and *egr1* were amplified from 24 hpf zebrafish embryos and cloned in pCS2 + vector, using respective restriction sites. The *ascl1a:*GFP*-luciferase, lin28a:*GFP*-luciferase, insm1a:*GFP*-luciferase*, *her4.1:*GFP*-luciferase*, and *mmp9:*GFP*-luciferase* constructs have been described previously [32–34]. *malat1* and *talam1* promoter (∼4 kb) was amplified from zebrafish genomic DNA and cloned into *pT2AL200R150G* vector, upstream of *gfp*, using primers harboring XhoI and SalI sites. *malat1* promoter was also cloned into the pEL luciferase expression vector using primers harboring XhoI and EcoRI sites, to generate *malat1:*GFP*-luciferase* construct.

Site-directed mutagenesis was performed to add *malat1* MO-binding site, upstream of GFP, on the pEGFP-N1 construct, using the protocol described in Ramachandran *et al.*, 2012 [34]. Templates for the *dlld* probe were amplified from 24 hpf embryos using primer pairs with a T3 polymerase binding site on the reverse primer, as listed in Supplementary Table S1. The *talam1* probe was designed by cloning the *malat1* splice site region into the pCS2 + vector using ClaI/StuI restriction sites, linearized with ClaI, and transcribed using SP6 polymerase.

### ChIP assay

Chromatin immunoprecipitation (ChIP) assays were conducted using a minimum of 10 adult zebrafish retinas or 6 mouse retinas at 2 days post-injury (dpi). Cells were lysed, and chromatin was sonicated to produce fragments ranging from 500 to 800 bp, following the method described by [35, 36]. The sonicated chromatin was divided into three equal portions: one served as the input control, another was immunoprecipitated using Rabbit polyclonal antibody against pSmad3 (Abcam, ab52903) or Rabbit polyclonal antibody against β-Catenin (Abcam, ab6302), and the third was immunoprecipitated with Rabbit IgG (Sigma–Aldrich, I5006), which served as a negative control. The primers used for the ChIP assays are detailed in Supplementary Table S1.

### Luciferase assay

For luciferase assays, single-cell zebrafish embryos were injected with ∼1 nl of a solution containing 0.02 pg of *Renilla* luciferase mRNA (normalization), 5 pg of a *promoter*:GFP-*luciferase* vector (*malat1*, *mmp9*, *ascl1a*, *lin28a*, *her4.1*, and*insm1a* promoter), and either *her4.1* mRNA, *her4.1* MO, *malat1* MO, SB431542 drug or Tgf-β1 protein, as required. After 24 h, embryos were divided into three groups (∼80 each) and lysed for dual-luciferase assays (Promega, E1910). For all luciferase assays, the values have been normalized to Renilla luciferase, which was co-injected in control and treatment conditions to serve as an internal standard, which compensated for potential variability in the transfection efficiency, embryo viability and number of cells per embryo.

### Quantitative real‐time RT-PCR, validation RT-PCR

Retinas were harvested after dark adaptation, and total RNA was extracted using TRIzol (Sigma). After DNase I (New England Biolabs, M0303L) treatment, double-stranded cDNA (complementary DNA) was synthesized from total RNA using a combination of random hexamers and oligo dT primers from cDNA synthesis kit (Thermo Fisher Scientific, K1622) or SuperScript-III (Thermo Fischer Scientific, 18080051). Quantitative real-time PCR was performed with at least six biological replicates, with each sample run in triplicate using the BIORAD SYBR mix on an Applied Biosystems real-time PCR system. Gene expression levels in control versus treated retinas were analyzed using the ΔΔ*C*t method and normalized to *β-actin* or *l24* mRNA levels. The sequence of primers used for qPCR is provided in Supplementary Table S1.

### Western blotting and antibodies

Western blotting was performed using a single retina per experimental sample, lysed in Laemmli buffer. The lysate was size-fractionated on a 12% acrylamide gel under denaturing conditions and transferred to an immunoblot polyvinylidene fluoride (PVDF) membrane (Bio-Rad, cat. no. 162-0177). The membrane was then probed with specific primary antibodies and HRP-conjugated secondary antibodies for chemiluminescence detection using Clarity Western ECL (Bio-Rad, cat. no. 170-5061). Primary antibodies used in the study are rabbit polyclonal antibodies against human ASCL1/MASH1 (Abcam, cat. no. ab74065), Rabbit polyclonal antibody against LIN28a (Cell Signalling Technologies, cat. no. 3978), rabbit polyclonal antibody against Sox2 (cat. no. ab59776; Abcam), Rabbit polyclonal antibody against pSmad3 (Abcam, ab52903), Anti pYAP1 (phospho S127) antibody (EP1675Y), Rabbit polyclonal antibody against zebrafish Hdac1 (Harrison *et al.*, 2011), Rabbit polyclonal antibody against H3K27ac (ab4729), and Rabbit monoclonal antibody against Pten (138G6) (Cell Signaling Technologies, 9559). Gapdh was used as a loading control. HRP-conjugated secondary antibodies used were described previously [33]. For all western blotting assays, 1–2 technical replicates were performed for each of the three biological replicates.

### BrdU/EdU labeling, tissue preparation, and immunofluorescence

BrdU labeling was done by immersing fish in 5 mM BrdU (Sigma) or injecting 10 μl of 20 mM BrdU solution intraperitoneally, 4 h before euthanasia and retina dissection, unless otherwise mentioned. EdU labeling was performed via intraperitoneal injection of 10 mM EdU solution as described earlier [29, 37]. Fish were anesthetized, eyes dissected, lenses removed, fixed in 4% paraformaldehyde, cryopreserved, and sectioned [38]. Immunofluorescence protocols and antibodies were as previously described [32, 33, 37, 39]. Rat monoclonal antibody against BrdU (Abcam, catalog number ab6326), Mouse monoclonal antibody against human proliferating cell nuclear antigen (PCNA; Santa Cruz, catalog number sc-25280), Mouse polyclonal antibody against HuD (Santa Cruz, catalog number sc-48421), and Goat polyclonal antibody against protein kinase C β1 (PKCβ1) (Santa Cruz, catalog number sc-209-G) were used. Secondary antibodies were conjugated to Alexa Fluor. EdU staining on cryosections was done with a Click-iT EdU Alexa Fluor 647 Imaging kit (Thermo Fisher Scientific, C10640). For all experiments, 3–6 retinae were used as biological replicates.

### RNA *in situ* hybridization

RNA *in situ* hybridization (ISH) was carried out on retinal sections using fluorescein- or digoxigenin-labeled complementary RNA probes (FL/DIG RNA Labeling Kit, Roche Diagnostics), following the protocol described in [40]. Fluorescence ISH was performed according to the manufacturer’s instructions (Thermo Fisher Scientific, catalog numbers: T20917, B40955, and B40953).

### Strand-specific cDNA synthesis and ss-qPCR

Strand-specific cDNA was synthesized using a specifically recognized *talam1* primer with an attached tag, following the manufacturer’s protocol for gene-specific cDNA synthesis (Thermo Fisher Scientific, K1622). The 5′-tagged *talam1* cDNA was subsequently amplified, with the tag sequence as the forward primer and a *talam1*-specific reverse primer. For ss-qPCR, equal amounts of RNA were used to synthesize the first strand of *talam1* cDNA, while a separate aliquot was used to generate total cDNA using random primers and oligo-dT primers. qPCR was performed to analyze *talam1* expression, with normalization against β-actin using the corresponding total cDNA aliquot.

### *let-7* quantification

Quantitation of *let7* was done using TaqMan microRNA probes (Applied Biosystems) as per the instruction manual. Total RNA was reverse transcribed employing stem-loop primers, and real-time PCR was conducted by using the TaqMan PCR Kit on Applied Biosystems 7300 system.

### FACS

GFP positive (GFP+) and GFP negative (GFP−) cells were separated from injured *1016tuba1a: GFP* retinas at 4 dpi using a standardized protocol briefly outlined in [41]. 4 dpi injured retinas (40 each) from *1016tuba1a: GFP* were dissected in L15 media, followed by controlled hyaluronidase and trypsin treatment to prepare a single-cell suspension. The cells were sorted using a BD FACS Aria Fusion high-speed cell sorter. Total RNA was isolated from the sorted cells, and the levels of specific transcripts were quantified using qPCR.

### RNA immunoprecipitation

Single-cell suspension of 2 dpi zebrafish retinae (20 retinae/sample) was prepared, crosslinked, and subjected to RIP following the protocol mentioned [42], with minor modifications. Protein A beads (40 μl) were incubated with anti-Hdac1 or anti-Ezh2 antibodies for 2 h, while 20 μl of beads treated with rabbit IgG served as a negative control. Following stringent washing, beads were incubated with the lysate for over 12 h at 4 degrees. After incubation and stringent washes, RNA was isolated from the aliquoted lysate, Rabbit IgG, anti-Hdac1/ anti-Ezh2 pulldown beads, negative control, and analyzed by PCR.

### Microscopy, cell counting, and statistical analysis

All slides after immunostaining or RNA ISH were mounted and examined using a Nikon Ni-E fluorescence microscope equipped with fluorescence optics and a Nikon A1 confocal imaging system. PCNA + and BrdU + cells were manually counted by observing fluorescence and ISH signals under a bright field. The cell counts were done specifically in the injury sites for appropriate quantification. For every experimental condition, six retinas were examined, and the counts were averaged. The most representative picture was chosen to show the results. Statistical analysis was performed using a non-parametric test, Mann–Whitney *U* test to compare between groups, using GraphPad Prism. Error bars in all histograms represent standard deviation (s.d.).

## Results

### The lncRNA *malat1* is differentially expressed in the regenerating retina

**Figure 1.**
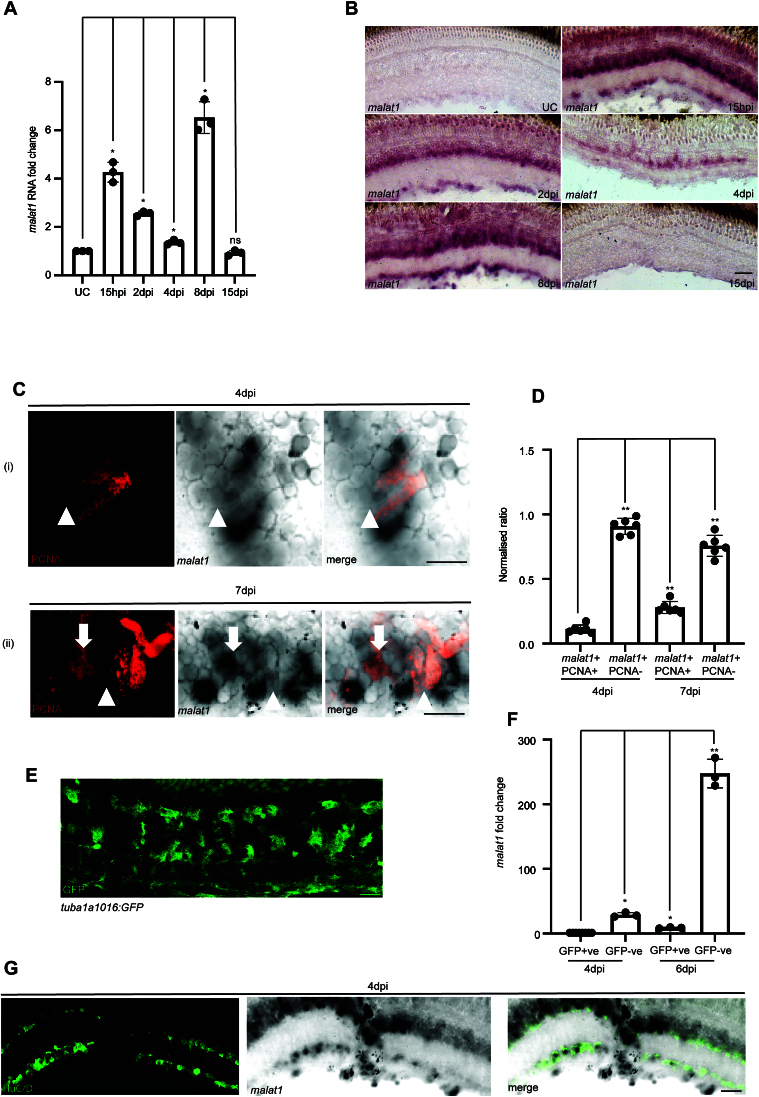
Temporal and spatial expression pattern of *malat1* during zebrafish retina regeneration. (**A**) qPCR analysis of *malat1* expression in the injured adult zebrafish retina at various time points post-injury. The data are presented as mean values ± standard deviation (SD), *P < 0.05; *n* = 3 biological replicates. (**B**) 20× bright-field (BF) images of retinal cross-sections showing *malat1* localization after ISH using a probe specific for zebrafish *malat1* in uninjured control (UC), 15 h post-injury (hpi), 2 days post-injury (2 dpi), 4 dpi, 8 dpi, and 15 dpi retinae. (**C**) 60× bright-field image of a retinal cross-section at 4 dpi, showing *malat1* expression excluded from most proliferating cells marked with PCNA. At 7 dpi, a higher proportion of proliferating cells expresses *malat1* compared to 4 dpi, indicating increased inclusion and quantified in (**D**); *P < 0.005; *n* = 6 biological replicates. Arrows indicate cells with *malat1* inclusion, while arrowheads indicate *malat1* exclusion. (**E**) A representative image of a retinal cross-section from the *tuba1016:gfp* transgenic line harvested at 4 dpi, with GFP fluorescence highlighting actively proliferating cells. (**F**) qPCR analysis of *malat1* enrichment in GFP Positive (actively proliferating) and GFP negative (neighboring) cells shows higher expression in neighboring cells compared to proliferating cells at 4 and 6 dpi. The data are shown as mean values ± SD, *P < 0.05, **P< 0.01; *n* = 3 biological replicates. (**G**) 20× confocal image of a retinal cross-section immunostained with HuC/D (amacrine cell marker) following zebrafish *malat1* ISH at 4dpi, demonstrating that a significant proportion of *malat1*^+^ cells are amacrine cells; ns indicates non-significant. The scale bar represents 10 μm in panels (B), (C), (E), and (G). Injury model: Mechanical needle poke.

We explored the transcript levels of zebrafish lncRNA (long non-coding RNA) *malat1* at different time points post-retinal injury by qPCR (Fig. 1A). *malat1* showed an upregulation in the reprogramming phase [15 h post-injury (hpi)] and late differentiation phase [8 days post-injury (dpi)], compared to its levels in the uninjured retina. The overall levels of zebrafish *malat1* expression is restored to uninjured levels at ∼15 dpi. This expression pattern is verified by the RNA ISH done on retinal cross-sections at various times post-injury (Fig. 1B). At 4 dpi, coinciding with the peak proliferation of retinal progenitors, zebrafish *malat1* levels were modestly elevated to those of the uninjured retina. A closer look at the RNA ISH at 4 dpi reveals that zebrafish *malat1* is absent in the proliferating BrdU-positive retinal progenitors while expressing in the immediately neighboring cells at 4 and 7 dpi (Fig. 1C and Supplementary Fig. S1A). Analysis of the proportion of proliferating *malat1*-positive cells, indicated by PCNA expression, revealed that at both 4 and 7 dpi, zebrafish *malat1* is predominantly located in cells adjacent to the PCNA-positive proliferating cells (Fig. 1D). However, by 7 dpi, the proportion of PCNA-positive cells expressing *malat1* increases. At 4 dpi, analysis of *malat1* expression using cell-type-specific markers showed that zebrafish *malat1* is primarily expressed in amacrine cells (Fig. 1G).

To further validate this observation, a *1016tuba1a:GFP* transgenic line, expressing GFP in proliferating retinal progenitors which have previously been characterized [38], was used, and FACS was employed to separate GFP-positive (GFP+) and GFP-negative (GFP−) cells. This allowed for assessing zebrafish *malat1* expression levels in GFP+ proliferating cells compared to GFP− cells (Fig. 1E). qPCR analysis of *malat1* in GFP-positive and GFP-negative cell populations revealed that zebrafish *malat1* is predominantly expressed in GFP-negative cells (Fig. 1F), confirming RNA ISH findings. Knockdown of zebrafish *malat1* using antisense MOs in early developmental stages causes developmental defects and high mortality rates, highlighting this lncRNA’s necessary roles (Supplementary Fig. S1B and C).

### Knockdown of *malat1* negatively affects retinal progenitor proliferation

**Figure 2.**
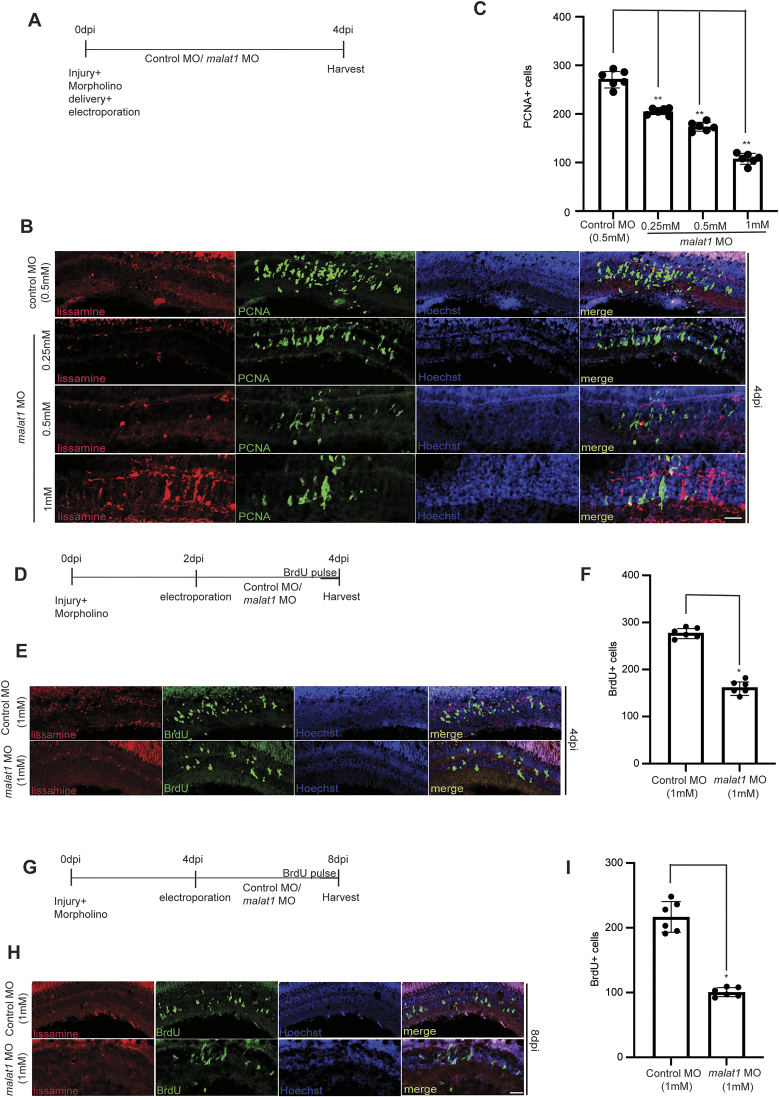
Impact of *malat1* knockdown on Müller glia-derived progenitor cell (MGPC) proliferation during different phases of zebrafish retina regeneration. *malat1* knockdown during proliferative, pre-proliferative, and late phases of retina regeneration results in reduced MGPC proliferation. A timeline schematic shows the sequence of injury, electroporation, and BrdU/EdU pulsing (4 h each), followed by tissue harvesting (**A**, **D**, and**G**). (**B**) Retina injured and electroporated with lissamine-tagged MO displayed a concentration-dependent decrease in PCNA+ cells when zebrafish *malat1* was knocked down from 0 to 4 dpi, compared to control. Quantification is shown in (**C**); *P < 0.001,**P< 0.0005; *n* = 6 biological replicates. (**E**) Knockdown of zebrafish *malat1* from 2 to 4 dpi led to a reduction in BrdU+ cells, quantified in (**F**); *P < 0.005; *n* = 6 biological replicates. (**H**) Inhibition of zebrafish *malat1* during the late proliferative phase (4 to 8 dpi) resulted in decreased BrdU+ cells, with quantification in (**I**); *P < 0.001; *n* = 6 biological replicates. ns indicates non-significant. The data are shown as mean values ± SD. The scale bar represents 10 μm (B, E, and H). Injury model: Mechanical needle poke

The zebrafish *malat1*-targeting MO, with a tested efficacy (Supplementary Fig. S2A), which negatively affected embryonic survival, was used to electroporate the injured retina (Fig. 2A). The number of retinal progenitors declined up to 50% upon the *malat1* knockdown in a concentration-dependent manner in the 4 dpi retina (Fig. 2B and C). Since zebrafish *malat1* expression is high at 15 hpi and 2 dpi, a delayed knockdown strategy was implemented from 2 dpi onwards. This delayed knockdown produced a similar reduction in proliferation of retinal progenitors (Fig. 2D–F). We performed a late *malat1* knockdown from 4 to 8 dpi (the post-proliferative and differentiating phase) (Fig. 2G). Compared to the control, we saw a decline in the number of BrdU^+^ cells at 8 dpi because of the *malat1* knockdown (Fig. 2H). These results suggest that zebrafish *malat1* contributes to the induction and maintenance of retinal proliferation. We then explored the effect of overexpression of zebrafish *malat1* in the regenerating retina. Overexpression of *malat1* increased proliferating retinal progenitors, hinting at its pro-proliferative effect. This effect was notably reduced when *malat1* was knocked down (Supplementary Fig. S2B and C). These observations show that zebrafish *malat1* is essential for proper and adequate induction of retinal progenitors in the regenerating retina.

### *malat1* influences the expression of regeneration-associated genes

**Figure 3.**
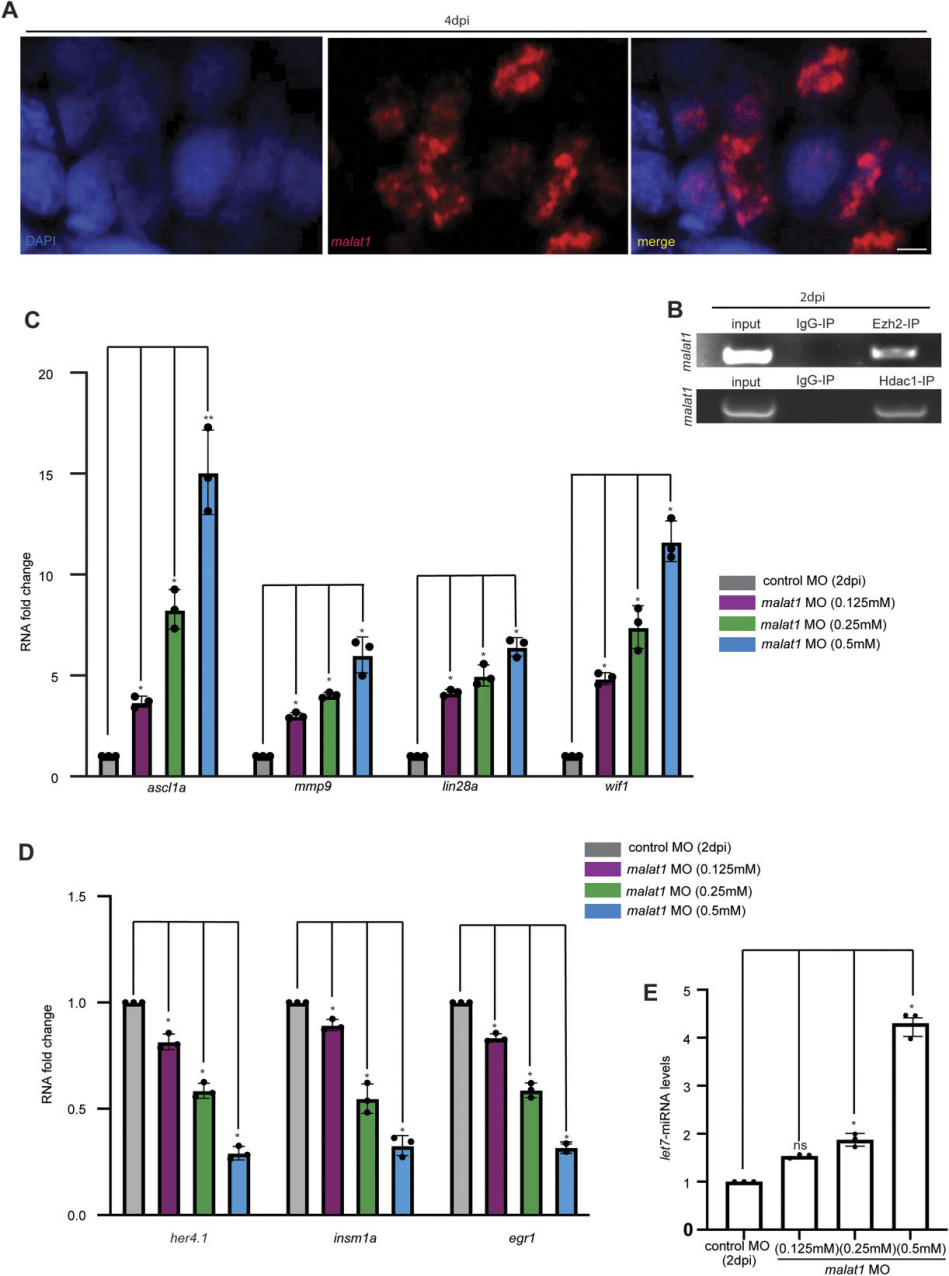
The *malat1* localization, interaction with other epigenetic modifiers, and regulation of different RAGs. (**A**) Confocal images of FISH showing zebrafish *malat1* localization in 4dpi retinal cross-sections after DAPI staining, indicating its presence at multiple nuclear loci; scale bar: 10 μm. (**B**) zebrafish *malat1* PCR check following RIP in 2 dpi retinal extract using anti-Ezh2 and anti-Hdac1 antibodies, with anti-IgG as a control, reveals a physical interaction between zebrafish *malat1* and these epigenetic modifiers. (**C**) qPCR analysis of various RAGs in zebrafish *malat1* knockdown retinae at 2 dpi shows increased expression of *ascl1a*, *mmp9*, *lin28a*, and *wif1*. The data are shown as mean ± standard deviation (SD), *P < 0.05; *n* = 3 biological replicates. (**D**) Conversely, a decrease in the expression of *her4.1*, *insm1a*, and *egr1* is observed in a concentration-dependent manner on zebrafish *malat1* knockdown. The data are shown as mean ± standard deviation (SD), *P < 0.04; *n* = 3 biological replicates. (**E**) qPCR analysis showing increase in *let-7* miRNA levels upon zebrafish *malat1* knockdown in 2 dpi retina in a concentration dependent manner. The data are shown as mean ± standard deviation (SD), *P < 0.05, **P< 0.01; *n* = 3 biological replicates. Injury model: Mechanical needle poke.

We observed the nuclear association of zebrafish *malat1* through fluorescence *in situ* hybridization (FISH) on retinal cross-sections, with DAPI (4',6-diamidino-2-phenylindole) marking the nuclei. This revealed a punctate nuclear expression pattern of zebrafish *malat1* (Fig. 3A). Given this distinctive punctate pattern, we hypothesized that *malat1* may exert a *trans* effect, meaning that it could influence distant genes or genomic regions, rather than acting locally at its site of expression. To explore this, we investigated zebrafish *malat1’s* potential association with epigenetic modifiers, including Ezh2, a catalytic component of the PRC2 complex, and Hdac1, a histone deacetylase. We performed RNA immunoprecipitation (RIP) of 2 dpi retinal extracts using anti-Ezh2 and anti-Hdac1 antibodies and probed for zebrafish *malat1*. The results showed that *malat1* was associated with Ezh2 and Hdac1 (Fig. 3B). The RIP assay supported the potential of zebrafish *malat1* in transcriptionally regulating gene expressions through its interaction with epigenetic modifiers. To investigate the potential regulatory role of zebrafish *malat1* in gene expression, we examined the expression patterns of several RAGs following *malat1* knockdown in zebrafish retina. Genes such as *ascl1a*, *mmp9*, *lin28a*, and *wif1* were found to be upregulated in the zebrafish following *malat1* knockdown at 2 dpi (Fig. 3C). In contrast, other genes involved in cellular proliferation, including *her4.1*, *insm1a*, and *egr1*, showed a dose-dependent decrease in expression with increasing *malat1*-MO concentration (Fig. 3D). To further confirm these findings, we performed a luciferase assay to assess the promoter activity of *ascl1a*, *lin28a*, *mmp9*, *her4.1*, and *insm1a* in zebrafish embryos. The results indicated that these genes were dysregulated following *malat1* knockdown (Supplementary Fig. S3A). Interestingly, opposite regulation of *ascl1a*,*mmp9*, and *egr1* was observed when zebrafish *malat1* was overexpressed (Supplementary Fig. S3B and B’). Additionally, several RAGs and epigenetic modifiers showed a decline in protein levels in the zebrafish *malat1* knockdown retina at 2 dpi (Supplementary Fig. S3C), which is also quantified (Supplementary Fig. S3D). This reduction in protein levels may be due to interference with mRNA translation by miRNAs like *let7*, which was shown to be elevated upon zebrafish *malat1* knockdown (Fig. 3E), potentially explaining the discrepancy between RNA and protein levels.

### Egr1 is a potential effector of *malat1* in inducing retinal progenitor proliferation

Given the syntenic conservation of many lncRNAs, we investigated genes near the *malat1* locus. Noting the proximity of *egr1* (Supplementary Fig. S3E), we examined its role in retina regeneration and explored its temporal expression pattern post-retinal injury. Previous studies have shown that Egr1 is a downstream effector in FGF2 and Notch signaling pathways mediated reprogramming of Müller glia into progenitor-like cells in the avian retina [43], suggesting an important role it plays in retinal regenerative processes. Expression of *egr1* peaks at 16 hpi during Müller glia reprogramming and declines to uninjured levels by 8 dpi (Supplementary Fig. S4A). Knockdown of *egr1* in *1016tuba1a:GFP* transgenic retinas showed a dose-dependent reduction in progenitor cell numbers at 4 dpi (Supplementary Fig. S4B and C). Knockdown and overexpression of *egr1* resulted in decreased and increased zebrafish *malat1* levels, respectively (Supplementary Fig. S4D and E), suggesting their interdependence during retina regeneration. The observed downregulation of zebrafish *malat1* upon *egr1* knockdown may be an indirect effect, potentially due to the increased expression of *her4.1*, a key effector of Notch signaling (Supplementary Fig. S4F). The regulatory influence of *her4.1* on zebrafish *malat1* expression is investigated in detail in the subsequent sections of this study.

**Figure 4.**
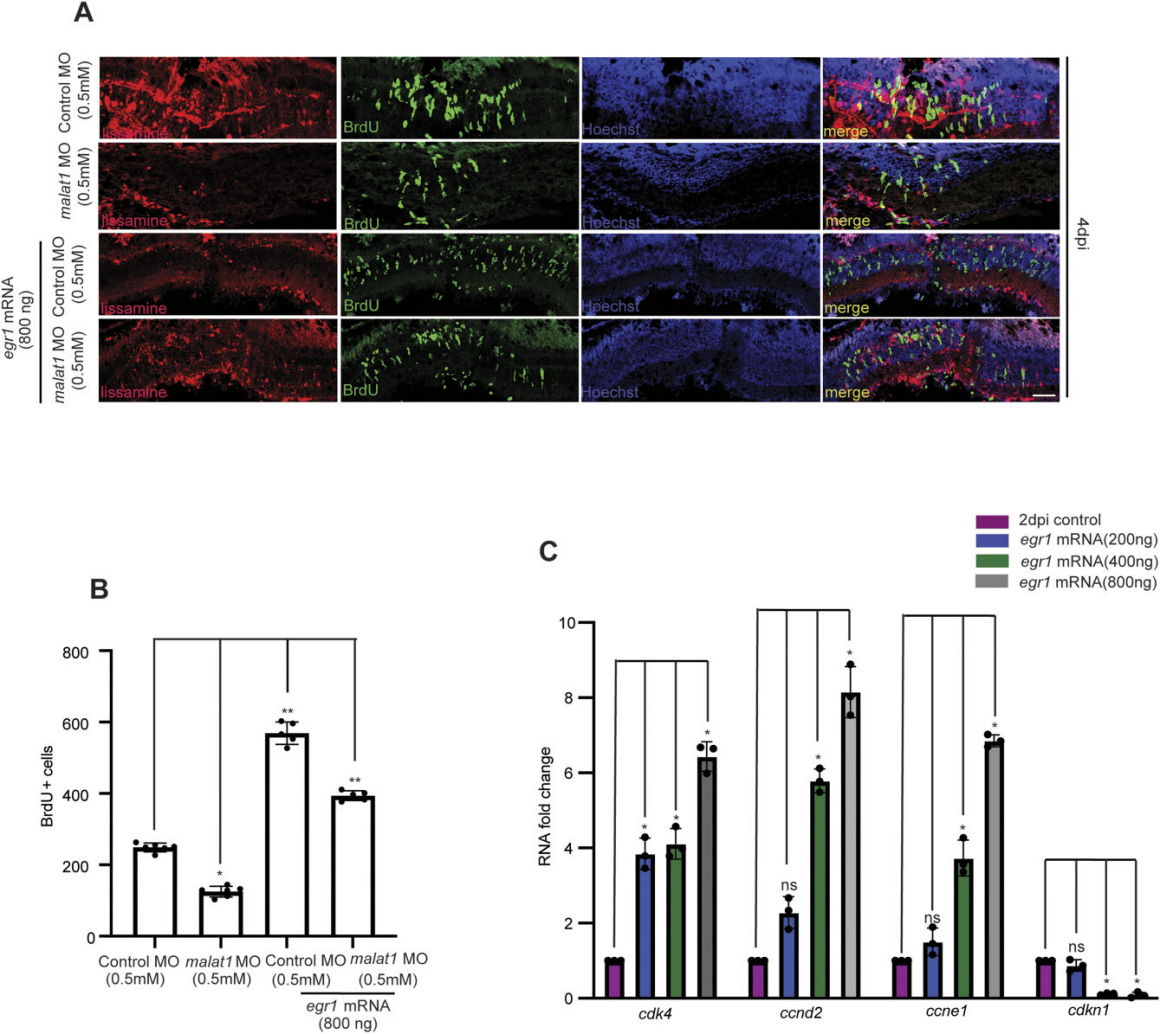
*malat1* knockdown effect is rescued by *egr1* overexpression and regulates cell cycle genes in MGPCs. (**A**) Confocal microscopy images show that the inhibitory effect of zebrafish *malat1* knockdown on MGPC proliferation is reversed by overexpression of *egr1*, which is quantified in (**B**). The data are presented as mean ± standard deviation (SD), *P < 0.0001; *n*= 6 biological replicates. This suggests that Egr1 partially mediates the activity of *malat1*. (**C**) qPCR analysis of cell cycle-related genes *cdk4*, *ccnd2*, *ccne1*, and *cdkn1* in the context of *egr1* overexpression shows significant deregulation of these genes. These changes in gene expression led to alterations in the proliferative capacity of MGPCs, highlighting the role of Egr1 in regulating cell cycle progression during zebrafish retina regeneration. The data are presented as mean ± standard deviation (SD), *P < 0.05, **P < 0.01; *n* = 3 biological replicates. ns indicates non-significant. The scale bar represents 10 μm (**A**). Injury model: Mechanical needle poke.

We hypothesized that the positive correlation between *malat1* and *egr1* expression plays a role in retinal progenitor proliferation. Transfecting injured retinas with *egr1* mRNA doubled progenitor proliferation. Notably, transfecting *egr1* mRNA alongside *malat1* MO rescued the decline in progenitor numbers caused by *malat1* knockdown at 4 dpi (Fig. 4A and B). These findings suggest that *malat1* influences retinal progenitors through Egr1, at least partially. qPCR analysis of cell-cycle regulators, including *cdk4*, *ccnd2*, *ccne1*, and *cdkn1*, showed that Egr1 promotes cell cycle progression, accounting for the increased progenitor numbers in *egr1* mRNA-transfected retinas (Fig. 4C).

### Egr1 positively influences retina regeneration through RAGs

**Figure 5.**
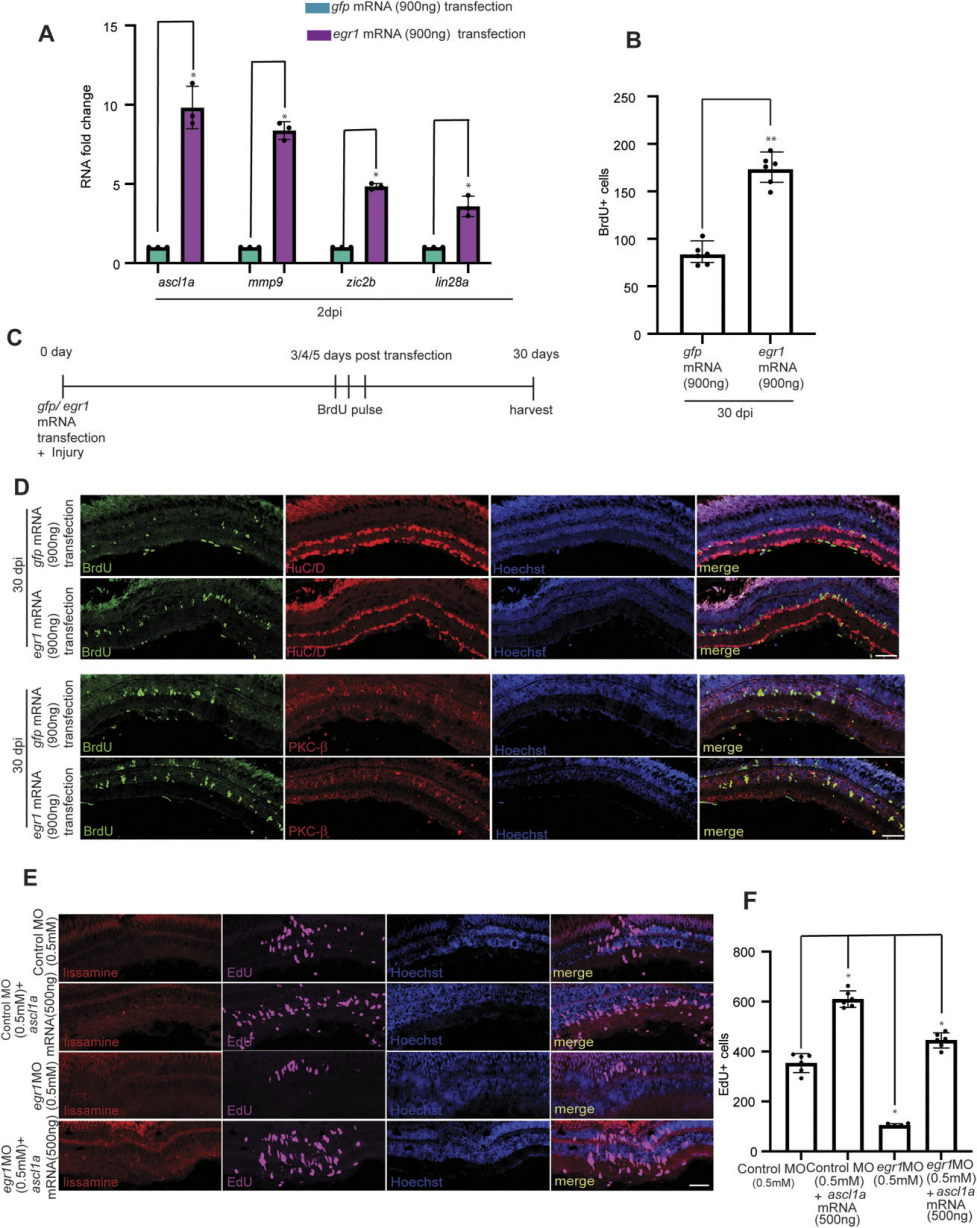
*egr1* overexpression enhances the proliferation, and the proliferated cells remain functional. Ascl1a requires Egr1 to mediate the regenerative response fully. (**A**) qPCR analysis at 2 dpi reveals that *egr1* overexpression enhanced the expression of RAGs like *ascl1a, mmp9, zic2b*, and *lin28a* in response to retinal injury in zebrafish. The data are presented as mean ± standard deviation (SD), *P < 0.05; *n* = 3 biological replicates. (**B**) Cells that incorporated BrdU at 3/4/5 dpi remained viable till 30 dpi. As found in Quantification. *P < 0.0005; *n* = 6 biological replicates. By 30 dpi, the proliferated cells (BrdU+) differentiated into HuD+ (amacrine cells) and PKC-β+ (bipolar cells), confirming they differentiated into different functional cell types. (**C**) Schematic representation of the experimental design. (**D**) Confocal images of retinal cross-sections at 30 dpi showing increased numbers of proliferated cells (BrdU +) following *egr1* overexpression compared to controls. (**E**) Confocal images of retinal cross-sections showing that knockdown of *egr1* in the context of *ascl1a* overexpression leads to a reduction in EdU + cells compared to *ascl1a* overexpression alone, indicating that Ascl1a and Egr1 act together to promote an effective regenerative response in zebrafish retina. Quantification is shown in (**F**); *P < 0.0005, **P < 0.0001; *n* = 6 biological replicates. Error bars represent standard deviation. Scale bars: 10 μm (D and E). Injury model: Mechanical needle poke.

Building on its effect on cell cycle progression, we explored if the Egr1 could influence the RAGs. The *egr1* mRNA transfected injured retina exhibited an enhanced expression of RAGs such as *ascl1a, mmp9, zic2b*, and *lin28a* (Fig. 5A). The *egr1* mRNA transfection in the injured retina caused an abundance of retinal progenitor formation, traced up to 30 dpi, evidenced by the survival of proliferating cells marked with BrdU at 3rd, 4th, and 5th dpi (Fig. 5DB). These progenitors could also differentiate into amacrine and bipolar retina cells (Fig. 5B–D). When transfected *in vivo* into zebrafish retina, *egr1* mRNA induced cell proliferation even in the uninjured retina (Supplementary Fig. S5A and B), but the induction of RAGs like *ascl1a, mmp9, zic2b, and lin28a* were minimal (Supplementary Fig. S5C). BrdU-tracing of these proliferating cells showed no traceability by 30 days (Supplementary Fig. S5D–F), suggesting that while Egr1 promotes proliferation, injury-induced upregulation of RAGs is necessary for enough progenitor formation and its viability. This prompted us to investigate the cooperation between Egr1 and a crucial RAG, Ascl1a, to promote effective retina regeneration. To test this hypothesis, we explored the influence of *ascl1a* overexpression on progenitor proliferation in an injured retina, with or without *egr1* knockdown. Interestingly, even with *ascl1a* overexpression, we saw a decline in the retinal progenitor proliferation when *egr1* is downregulated (Fig. 5E). Notably, despite a decrease in the retinal progenitor proliferation, the *ascl1a* overexpressed, and Egr1 declined retina had a higher number of retinal progenitors than that of *egr1* knockdown alone (Fig. 5F). Importantly, *ascl1a* overexpression did not significantly affect *egr1* levels (Supplementary Fig. S5G), supporting the idea that these factors function independently, through parallel pathways, but are both essential for promoting retinal progenitor formation.

### Egr1 is an effector of Wnt signaling in the regenerating retina

**Figure 6.**
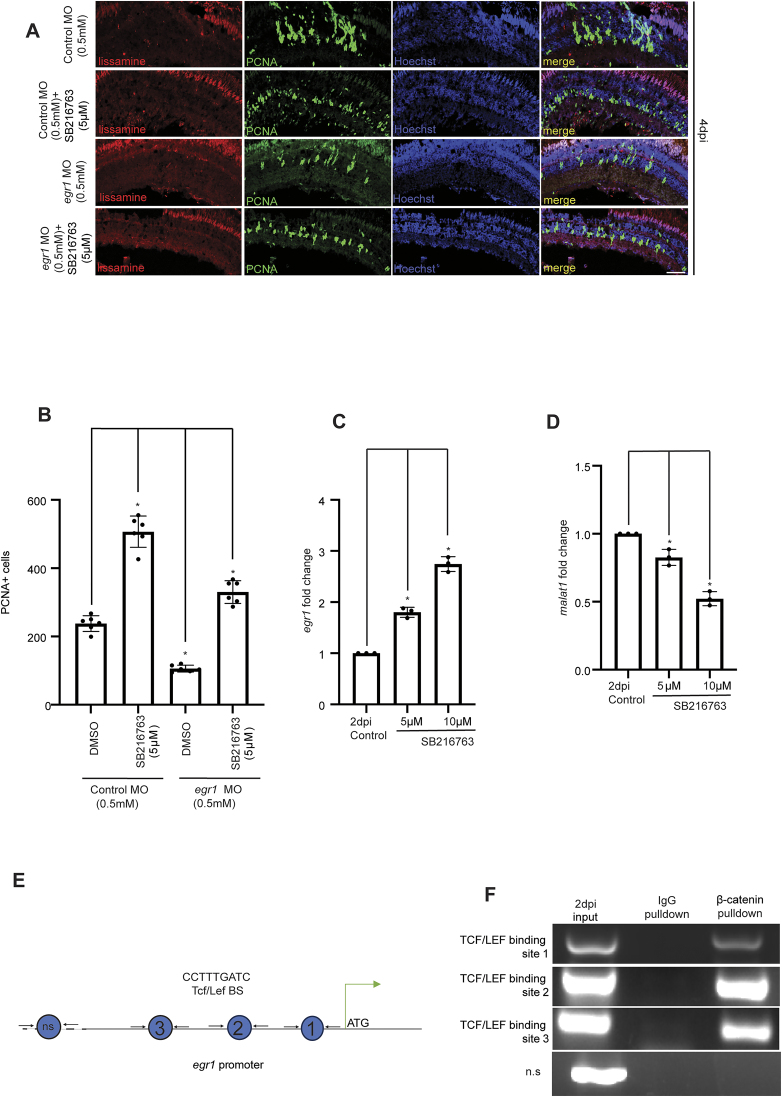
Wnt signaling mediates its effect by inducing *egr1*. (**A**) Confocal images of retinal cross-sections reveal that the increase in PCNA + cells induced by β-catenin stabilization through SB216763 drug administration was reduced following *egr1* knockdown in zebrafish retina. It has been quantified in (**B**). It hints that Wnt signaling may partly regulate MGPC proliferation by modulating *egr1* levels. The data are presented as mean ± standard deviation (SD), *P < 0.003; *n* = 6 biological replicates. Scale bars: 10 μm. (**C**) qPCR analysis demonstrates that *egr1* expression is significantly induced by stabilizing β-catenin (SB216763 administration), indicating that Wnt signaling plays a crucial role in regulating *egr1* expression during retinal regeneration. The data are presented as mean ± standard deviation (SD), *P < 0.05; *n* = 3 biological replicates. (**D**) qPCR showing zebrafish *malat1* levels get reduced on stabilizing β-catenin (SB216763 administration), *P < 0.05; *n* = 3 biological replicates. (**E**) Schematic representation of the TCF/LEF binding site (β-catenin binding site) on the *egr1* promoter. (**F**) PCR analysis following ChIP with anti-β-catenin antibody confirms that β-catenin directly binds to the *egr1* promoter to drive its transcription. ns indicates a non-specific site in ChIP PCR. Injury model: Mechanical needle poke.

Wnt signaling is an important gene regulatory pathway that is shown to be essential during retina regeneration [41]. We were intrigued to explore whether Egr1 contributes as an effector of Wnt signaling in inducing retinal progenitors. We treated zebrafish retinas with SB216763, a GSK3β inhibitor that stabilizes β-catenin and activates Wnt signaling, with or without *egr1* MO, and observed its effect on proliferation at 4 dpi (Fig. 6A). SB216763 treatment increased retinal progenitors, but this effect was alleviated when *egr1* was knocked down (Fig. 6A and B). The qPCR analysis of *egr1* mRNA in SB216763-treated retina revealed a dose-dependent increase in its expression levels at 2 dpi (Fig. 6C). However, the SB216763 drug treatment caused a decrease in the *malat1* expression (Fig. 6D), which suggested the possible direct regulation of *egr1* mRNA through Wnt signaling. To test this possibility, we analyzed the regulatory DNA elements of the *egr1* promoter for potential TCF/LEF (T cell factor/lymphoid enhancer factor)-binding sites. We identified three TCF/LEF-binding sites immediately upstream of the *egr1* gene in zebrafish (Fig. 6E). A ChIP assay at 2 dpi using a β-catenin antibody showed occupancy of β-catenin to these sites on the *egr1* promoter (Fig. 6F), supporting that Wnt signaling directly regulates Egr1 expression during retina regeneration.

To further verify it, we treated injured retinas with XAV939, a Wnt signaling inhibitor, with or without *egr1* overexpression (Supplementary Fig. S6A). While XAV939 treatment reduced retinal progenitor numbers, *egr1* overexpression rescued the progenitor count to control levels (Supplementary Fig. S6A and B). These observations suggest that Wnt signaling influences the induction of retinal progenitors through Egr1 and that the blockade of Wnt signaling could be rescued through *egr1* overexpression. The qPCR analysis of the *egr1* mRNA in SB216763 and XAV939 treated conditions reveals a positive interplay between Wnt signaling and Egr1 (Fig. 6C and Supplementary Fig. S6C). This suggests that Wnt signaling regulates retinal progenitor induction through Egr1.

### *malat1* is suppressed by Delta-Notch signaling and functions to induce *deltaD*

**Figure 7.**
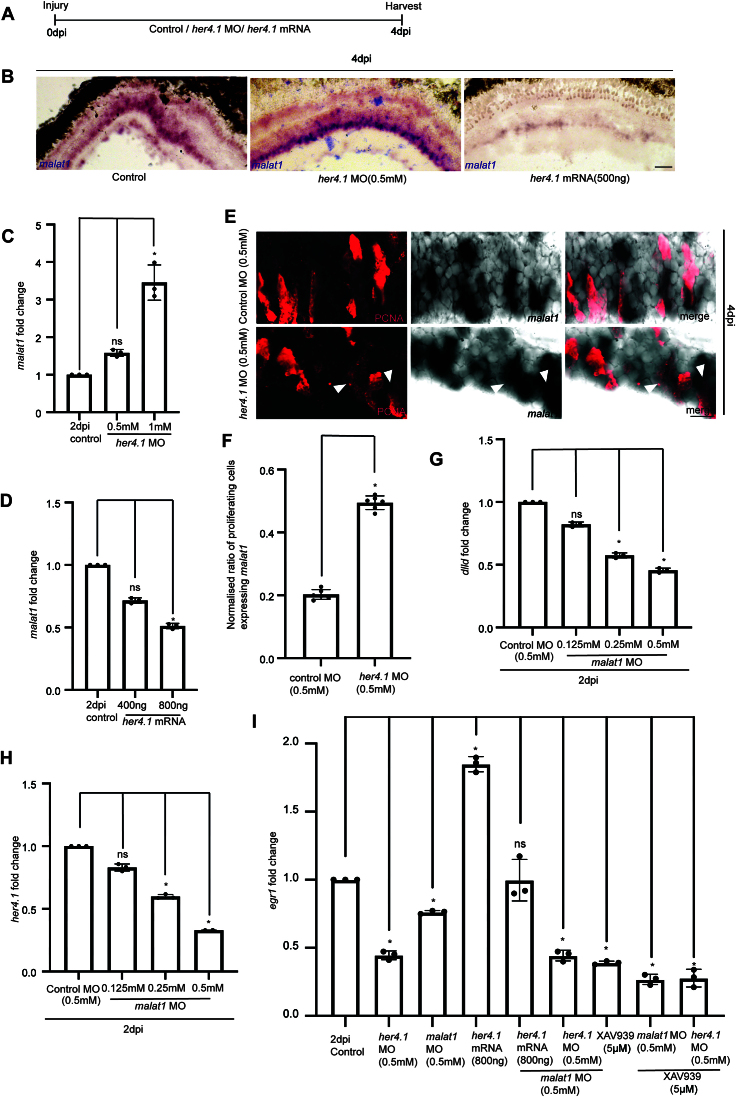
*malat1* regulates *egr1* in a cell non-autonomous manner via Notch signaling. Wnt signaling and Notch signaling synergistically regulate *egr1* levels. (**A**) Experimental timeline schematic. (**B**) Bright-field image of retinal cross-sections showing zebrafish *malat1*ISH after *her4.1* knockdown and overexpression respectively, compared to control in zebrafish retina. (**C**) qPCR analysis of zebrafish *malat1* levels on *her 4.1* knockdown, *P < 0.05; *n* = 3 biological replicates, and (**D**) *her 4.1* overexpression, *P < 0.05; *n* = 3 biological replicates. The data are represented as mean ± standard deviation (SD). (**E**) 60× confocal images showing increased inclusion of zebrafish *malat1* expression within proliferating cells following *her4.1* knockdown compared to control in zebrafish retina, quantified in (**F**). The data are presented as mean ± standard deviation (SD), *P < 0.003; *n* = 6 biological replicates. Arrowheads indicate *malat1* localization in proliferating cells. This observation suggests that *her4.1* suppresses *malat1* expression in these cells. (**G**) qPCR analysis showing the impact of *malat1* knockdown on *dlld* expression, *P < 0.05; *n* = 3 biological replicates, and *her4.1* expression (**H**), *P < 0.05; *n* = 3 biological replicates. The data are presented as mean ± standard deviation (SD). (**I**) qPCR analysis shows that combined knockdown of *her4.1* and *malat1* reduces *egr1* levels to the same extent as *her4.1* knockdown alone, suggesting that *malat1* regulates *egr1* through *her4.1* (Notch signaling). Combined inhibition of Wnt signaling (XAV939) and *malat1* knockdown or *her4.1* knockdown caused an even greater decrease in *egr1* expression. This suggests that *malat1* regulates *egr1* levels in proliferating cells, through Notch signaling, while Notch and Wnt signaling pathways independently regulate *egr1* levels. The data are represented as mean ± standard deviation (SD), *P < 0.05; *n* = 3 biological replicates. Scale bars represent 10 μm in (B) and (E). ns indicates non-significant. Injury model: Mechanical needle poke.

Delta-Notch signaling plays a dynamic and context-dependent function in zebrafish retina regeneration. Suppression of Delta-Notch signaling has been reported by some to increase the regenerative zone by enabling more Müller glia to re-enter the cell cycle [39], and others highlight its function in maintaining quiescence in Muller glia via Notch3/DeltaB signaling [44]. Conversely, one of the downstream targets of Notch signaling like Her4.1 have been reported to maintain the proliferative status of retinal progenitors [45]. Also, Notch1 activity has been shown to be required to sustain progenitor proliferation during mammalian retinal development [46]. Collectively, these results indicate that the regenerative outcome of Notch signaling are extremely context-dependent and are likely to be influenced by particular ligand–receptor interactions. We investigated whether Her4.1 affects the *malat1* expression. For this, we either downregulated or overexpressed *her4.1* in the injured retina and checked for the zebrafish *malat1* levels (Fig. 7A). Knockdown of *her4.1* increased (Fig. 7B and C), while its overexpression reduced the zebrafish *malat1* expression (Fig. 7B and D). Notably, the typical exclusion of *malat1* expression from cells that are proliferating in the regenerating retina was significantly disrupted, as *malat1* was found to be expressed in the proliferating cells when *her4.1* was downregulated (Fig. 7E and F). We further explored the zebrafish *malat1* promoter activity in the presence of *her4.1*-targeting MO or its mRNA using luciferase assay in zebrafish embryos. We saw a negative regulation of the *malat1* promoter activity by Her4.1 (Supplementary Fig. S7A). These results suggest that the regulation of *malat1* is mediated through Delta-Notch signaling component, Her4.1, mainly to restrict its expression to the cells neighboring to the proliferating progenitors.

FISH was conducted using antisense probes for *malat1*, *dlld* (DeltaD encoding transcript), and *her4.1* mRNA. Co-labeling of *malat1* and *dlld* was observed (Supplementary Fig. S7B), while *malat1* and *her4.1* showed mutual exclusion in the retina at 4 dpi (Supplementary Fig. S7C). A cell-sorting analysis of *1016tuba1a*:GFP transgenic retina at 4dpi confirmed the abundance of *dlld* in GFP negative and *her4.1* expression in GFP positive fraction (Supplementary Fig. S7D). The *her4.1* expression is a feature of proliferating retinal progenitors [29]. These findings prompted us to explore whether *malat1* expression in the neighboring cells influenced Delta-Notch signaling. We speculated if the *malat1* could influence the expression of Delta ligand, leading to the activation of Notch receptors in the proliferating retinal progenitors. The *malat1* knockdown caused a decline in the *dlld* mRNA in a dose-dependent manner at 2 dpi (Fig. 7G). This decline in the *dlld* was also accompanied by a decrease in the *her4.1* mRNA levels (Fig. 7H). This suggests that *malat1* regulates Notch signaling by affecting *dlld* expression, eventually influencing *her4.1* levels in proliferating cells.

Initially, we hypothesized that *malat1* regulated *egr1* in a *cis* manner, implying that both should be expressed in the same cell. However, given that Wnt signaling is active in proliferating cells (Meyers *et al.*, 2012; Ramachandran *et al.*, 2011) and promotes *egr1* expression, which pushes cells to enter into the cycling phase, we reassessed the localization of *egr1*. Cell-sorting analysis of *1016tuba1a*:GFP transgenic retina at 4 dpi confirmed the abundance of *egr1* in GFP positive fraction (Supplementary Fig. S7E). It suggests that zebrafish *malat1* may regulate *egr1* non-cell autonomously, potentially through juxtacrine signaling mechanisms like Notch signaling. Knockdown experiments demonstrated that both *her4.1* and *malat1* downregulate *egr1*, but the effect of combined knockdown mirrored that of *her4.1* knockdown alone, suggesting that *malat1* acts on *egr1* primarily through *her4.1*. Additionally, inhibiting Wnt signaling using XAV939 alongside *malat1* knockdown produced a synergistic reduction in *egr1* (Fig. 7I), highlighting the independent contributions of these pathways in inducing retinal progenitors.

### TGF-β signaling suppresses stable *malat1* expression in zebrafish but enhances *Malat1* expression in mice

**Figure 8.**
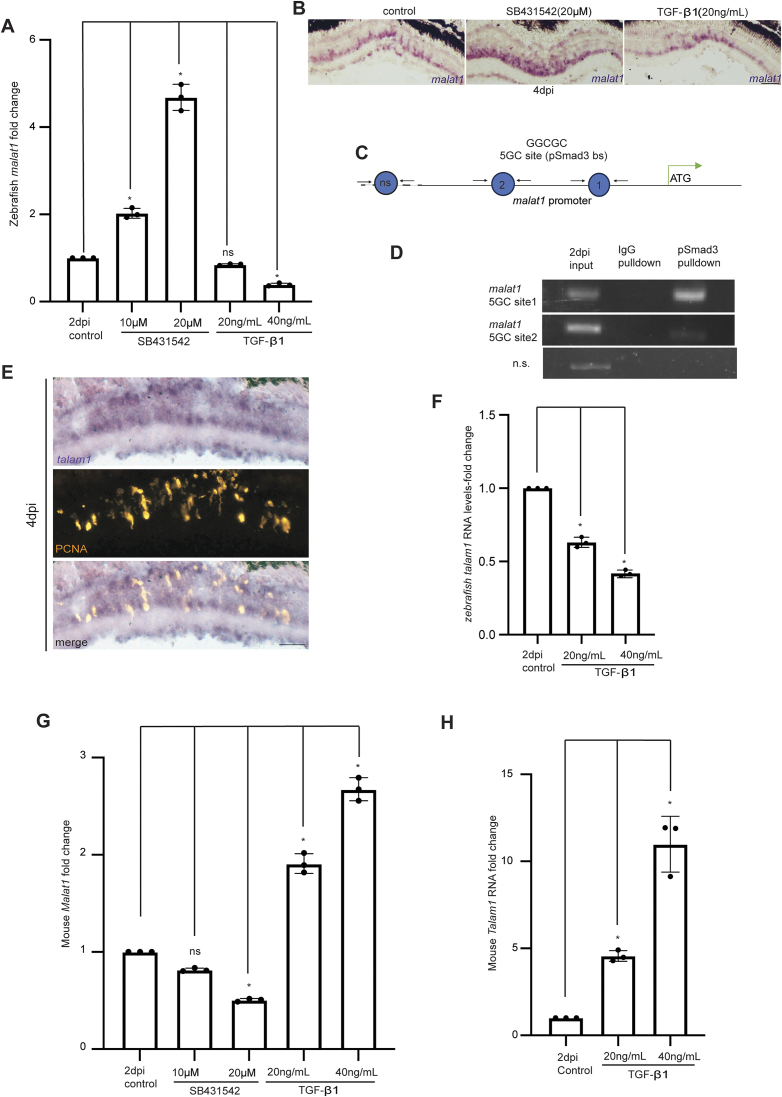
Differential regulation of *malat1* by TGF-β signaling in zebrafish and mice, and the interplay of *talam1* in mediating this regulation. (**A**) qPCR analysis of zebrafish *malat1* levels following inhibition and overexpression of TGF-β signaling in zebrafish retina. The data are presented as mean ± standard deviation (SD), *P < 0.05; *n* = 3 biological replicates. (**B**) Bright-field images of retinal cross-sections following *malat1*ISH in zebrafish, treated with SB431542 (TGF-β inhibitor) and in TGF-β1 overexpression scenarios, revealing its repressive role on zebrafish *malat1* expression. (**C**) Diagrammatic representation of 5GC elements on the *malat1* promoter in zebrafish, illustrating the potential regulatory sites for TGF-β signaling. (**D**) PCR analysis after pSmad3 ChIP in 2 dpi zebrafish retina shows that pSmad3 occupies 5GC elements on the zebrafish *malat1* promoter. IgG pulldown serves as a negative control, revealing the occupancy of pSmad3 on 5GC elements of the zebrafish *malat1* promoter. (**E**) 20× bright-field (BF) images of retinal cross-sections showing distinct *talam1* expression after ISH using a probe specific for *talam1* in 4 dpi zebrafish retina. (**F**) ss-qPCR analysis in TGF-β1 overexpression scenarios showing downregulation of zebrafish *talam1*, indicating the inhibitory role of TGF-β signaling on *talam1* during zebrafish retina regeneration. The data are presented as mean ± standard deviation (SD), *P < 0.05; *n* = 3 biological replicates. (**G**) qPCR analysis of mouse *Malat1* levels following inhibition and overexpression of TGF-β signaling in injured mouse retinae. The data are presented as mean ± standard deviation (SD), *P < 0.05; *n* = 3 biological replicates. (**H**) ss-qPCR analysis showing that mouse *Talam1* is upregulated in the injured mouse retina following TGF-β1 overexpression. The data are presented as mean ± standard deviation (SD), *P < 0.05; *n* = 3 biological replicates. The scale bar is 10 μm in (B) and (E). ns indicate non-significant. Injury model: NMDA injury.

Tgf-β signaling is one of the major pro-proliferative pathways essential for zebrafish retina regeneration [47]. To keep the injury method consistent across species, NMDA was injected via the cornea to induce retinal damage in both zebrafish and mice. We explored whether Tgf-β signaling plays a role in regulating *malat1* expression. Inhibition of Tgf-β signaling in the regenerating retina using the blocker SB431542 caused an elevated expression of zebrafish *malat1* in a dose-dependent manner (Fig. 8A). Conversely, the Tgf-β1 protein inhibited zebrafish *malat1* expression (Fig. 8A). The *malat1* RNA ISH performed in 4 dpi retina (Fig. 8B) and the luciferase assay performed in zebrafish embryos using zebrafish *malat1* promoter constructs (Supplementary Fig. S8A) confirmed these results. A closer look at the regulatory DNA elements of the zebrafish *malat1* gene revealed two 5GC elements, which are putative pSmad3 binding sites essential for transcriptional activation by Tgf-β signaling [48] (Fig. 8C). A ChIP assay performed in 2 dpi retinal protein extracts using the pSmad3 antibody confirmed that pSmad3 occupied these 5GC elements (Fig. 8D). However, despite this binding, qPCR, RNA ISH, and luciferase assays demonstrated that TGF-β signaling represses zebrafish *malat1* expression, presenting an apparent paradox. This contradiction implies that there is another regulatory pathway affecting zebrafish *malat1* expression, prompting us to explore the function of *talam1*, an antisense transcript of *malat1*

Previous studies have indicated that human *TALAM1*, an antisense transcript of human *MALAT1*, is essential for the maturation of the *MALAT1* transcript. Mature *MALAT1* has improved half-life and is functional [49, 50]. However, the existence of *talam1* in zebrafish had not been reported. To determine whether *talam1* is expressed in zebrafish, we performed strand-specific RT-PCR in zebrafish embryos (Supplementary Fig. S8B). Having found its expression in zebrafish embryos, we created zebrafish transgenic lines driving GFP under the control of zebrafish *talam1* (Supplementary Fig. S8C) and *malat1* promoters (Supplementary Fig. S8D). While both transgenic lines exhibited overlapping GFP expression, they also displayed distinct expression patterns, suggesting that despite their genomic proximity, the zebrafish *talam1* and *malat1* promoters function autonomously and are independently regulated. The genomic location of both genes is on sense and antisense strands of the gene loci (Supplementary Fig. S8E). The RNA-ISH also confirmed the expression of *talam1* in 4 dpi zebrafish retina, reinforcing its involvement in the regenerative process (Fig. 8E). Although the dependence of *MALAT1* on *TALAM1* for its maturation and stability has been established in human cell lines, we reasoned that a similar regulatory mechanism might exist in zebrafish. Therefore, we sought to investigate how Tgf-β signaling influences *talam1* expression in the zebrafish retina. The promoter of zebrafish *talam1* harbored pSmad3-binding TIE elements (Supplementary Fig. S8F). TIE elements are sites wherein the pSmad3 binds with other protein factors to cause transcriptional repression [47, 51]. A ChIP assay performed in 2 dpi retinal protein extracts using pSmad3 antibody confirmed the occupancy of these TIE elements of zebrafish *talam1* promoter by pSmad3 (Supplementary Fig. S8G), suggesting that Tgf-β signaling directly represses zebrafish *talam1* transcription. Consistently, TGF-β1 protein delivery caused a decline in the expression levels of zebrafish *talam1* in the 2 dpi retina (Fig. 8F). This decline of zebrafish *talam1* levels could probably cause reduced mature and functional zebrafish *malat1* transcript levels in Tgf-β signaling-induced zebrafish retina.

In mammals, the TGF-β signaling is anti-proliferative except in cancerous conditions [52]. This raised the question of whether *malat1* regulation differs between zebrafish and mice during retinal regeneration. The mouse *Malat1* gene promoter analysis revealed the presence of both 5GC and TIE elements (Supplementary Fig. S8H). A ChIP assay performed using PSmad3 antibody in retinal protein extracts of 2 dpi mice retina revealed that 5GC sites as well as TIE elements were bound by PSmad3 (Supplementary Fig. S8I). However, in contrast to the zebrafish retina, inhibiting TGF-β signaling in the injured mice retina at 2 dpi downregulated mouse *Malat1* RNA levels (Fig. 8G). The TGF-β1 protein caused an upregulation of both mouse *Malat1* (Fig. 8G) and mouse *Talam1* (Fig. 8H) transcript levels in mice retinas at 2 dpi, suggesting a species-specific regulatory mechanism where TGF-β signaling stabilizes *Malat1* expression in mammals rather than repressing it.

**Figure 9.**
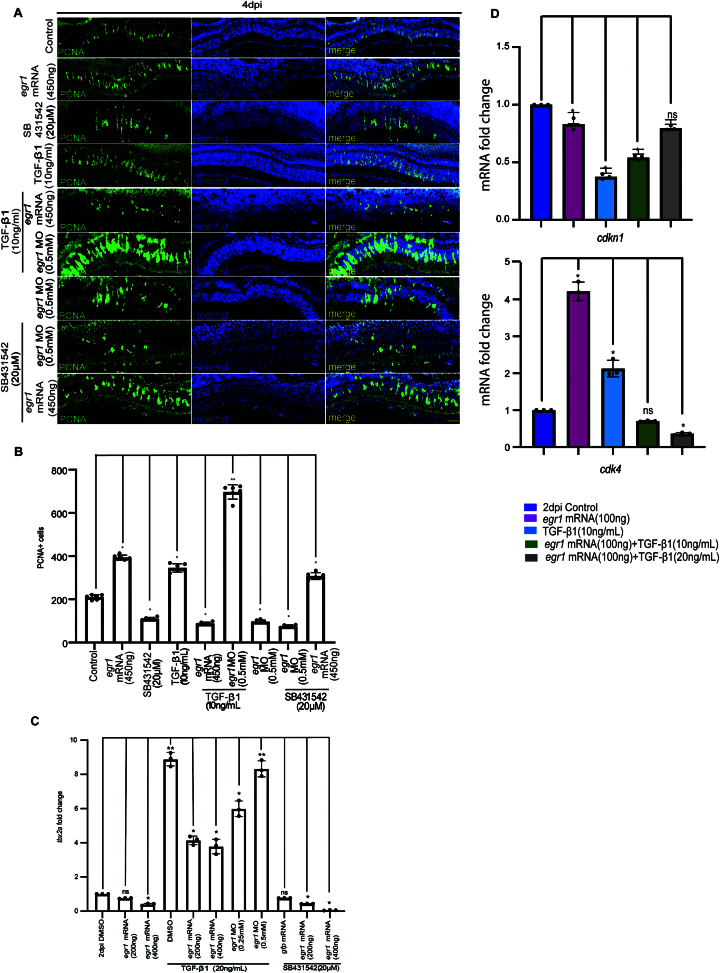
Impact of TGF-β signaling and Egr1 on proliferation and cell cycle regulation during retina regeneration. (**A**) Confocal images of retinal cross-sections at 4 dpi showing proliferation status upon modulating TGF-β signaling and Egr1 levels, individually and in combination. Scale bar: 10 μm. Quantification is shown in (**B**). The data reveal that TGF-β signaling and Egr1 individually promote proliferation, but their combined overexpression decreases PCNA + cells. The data are presented as mean ± standard deviation (SD), *P < 0.005; *n* = 6 biological replicates. (**C**) qPCR analysis of *tbx2a* expression at 2 dpi under conditions of TGF-β1 overexpression with or without *egr1* overexpression or knockdown, and TGF-β signaling inhibition (SB431542), alone or combined with *egr1* overexpression or knockdown in zebrafish retina. The data are presented as mean ± standard deviation (SD), *P < 0.05; *n* = 3 biological replicates. (**D**) qPCR analysis shows synergistically increased *cdkn1* expression (upper) and reduced *cdk4* expression levels (lower) when TGF-β1 and Egr1 are overexpressed together. This suggests that Egr1 may be sequestered away from its target sites under these conditions, altering cell cycle regulation. The data are presented as mean ± standard deviation (SD), *P < 0.05, **P< 0.01; *n* = 3 biological replicates. NS indicate non-significant. The scale bar is 10 μm in (**A**). Injury model: Mechanical needle poke.

We also found an anticipated decrease of zebrafish *egr1* mRNA levels (Supplementary Fig. S8J) and an increase in mouse *Egr1* mRNA levels in the retina (Supplementary Fig. S8K) because of TGF-β1 protein delivery. After zebrafish retinal injury, manipulating Tgf-β signaling and Egr1 expression revealed that co-overexpression of both caused a synergistic decrease in proliferation (Fig. 9A and 9B). Co-overexpression of TGF-β1 and *egr1* significantly elevated *tbx2a* levels, a pro-proliferative factor (Fig. 9C). Based on previous studies [53], there is a possible co-sequestration of Tbx2a and Egr1, negating both factors’ pro-proliferative effects. Decreased *cdk4* and increased *cdkn1* levels in the scenario supported the hypothesis that Egr1 cannot perform its function in the co-overexpression scenario (Fig. 9D). In mice, TGF-β signaling upregulates *Egr1*, which mirrors the co-overexpression scenario observed in zebrafish, potentially explaining the opposing effects of TGF-β signaling on proliferation in both species.

## Discussion

Retina regeneration is efficient in lower vertebrates such as fish and frogs. However, mammals do not have this ability, especially in central nervous system organs such as the retina. Manipulating gene expression pathways in the mouse retina, which zebrafish retina regeneration has mostly resulted in positive responses, albeit incomplete [54–56]. Several holistic gene expression studies have revealed that the mammalian retina exhibits some regenerative potential, though it remains incomplete. In this study, we tried to explore the importance of long non-coding RNA *malat1* during zebrafish retina regeneration. We also explored if *malat1* is differentially regulated in mice and zebrafish retina, which could probably account for the lack of complete regeneration in injured mice retina.

At first, we explored the importance of *malat1* during zebrafish development, as no phenotype was observed in mice after *Malat1* knock-out [25]. We saw a significant mortality of zebrafish embryos because of *malat1* knockdown, suggesting important gene expression events are guided through this lncRNA, unlike in mice. It is to be noted that despite normal development, the *Malat1* knock-out disrupted many adjacent genes of the *Malat1* locus [25]. A similar phenomenon could also be at play in zebrafish, as we found a significant decline in the retinal progenitor formation upon *malat1* knockdown in zebrafish retina at early and late time points.

The *malat1* RNA is nuclear localized in its expression, which indicates its genomic functions. The *Malat1* is shown to interact with nuclear proteins such as Nucleolin and Nucleophosmin [57]. The forced expulsion of *Malat1* from the nucleus causes dysregulation of TDP-43, whose aggregation is implicated in neurodegeneration [58]. Our study also reveals the interaction of *malat1* to histone deacetylase Hdac1 and histone methylase Ezh2 of polycomb repressor complex 2 in zebrafish retina during regeneration. These findings suggest the involvement of *malat1* in epigenetic regulation, possibly through interacting with epigenetic modifiers like Hdac1 and Ezh2, to regulate its access or activity on various chromatin loci. Although nuclear, a portion of the cytoplasmic localization of *Malat1* in the brain serves as a translatable coding RNA by synaptic stimulation [59].

*Malat1* is known for its role in diverse cell types and influence on other signaling pathways such as Hippo-Yap and Wnt signaling. The *MALAT1* level stays upregulated in diabetic cardiomyopathy. The knockdown of *MALAT1* in the diabetic cardiomyopathy mice model alleviates the clinical manifestations of high glucose on heart muscles, such as inflammation and collagen accumulation, by influencing the Hippo-Yap pathway [60]. *Malat1* is regulated epigenetically through histone demethylase JMJD2C at H3K9me3 and H3K36me3 locations of the *Malat1* promoter, causing its upregulation and subsequent activation of Wnt signaling in cancer metastasis [61]. These scenarios prompted us to explore the expression dynamics of *malat1* during retina regeneration and its implications in downstream gene expressions and signaling pathways.

The physical proximity of one of the pro-mitotic genes, *egr1*, to the *malat1* locus in linear DNA was unique to zebrafish when compared to humans and mice. When exploring *egr1* levels in *malat1* knockdown, we saw a dose-dependent decrease in *egr1* mRNA levels. The knockdown of *egr1* also caused a decline in retinal progenitors. Notably, *egr1* is involved in retinal development during zebrafish embryogenesis, as its knockdown impaired eye formation, highlighting its key role in retinal cell proliferation and fate [28]. Interestingly, the declining retinal progenitor number because of the zebrafish *malat1* knockdown could be rescued by *egr1* overexpression. This finding suggests that one of the regulatory pathways governed by zebrafish *malat1* is via Egr1. It is also important to note that several RAGs, such as *ascl1a*, *sox2*, *lin28a*, *hdac1*, and *yap1*, were deregulated because of *malat1* knockdown. It was intriguing to note that the pSmad3 protein, an effector of Tgf-β signaling, was upregulated in the *malat1* knockdown retina. This observation was indicative of an interrelationship between Tgf-β signaling and *malat1*.

The Retinal progenitor proliferation is strongly influenced by Wnt signaling during retina regeneration in zebrafish [41]. The Wnt signaling could also influence mammalian retinal neuronal reprogramming and regeneration [56, 62]. Pharmacological stabilization of β-catenin enhanced retinal progenitor formation in the injured retina and could facilitate retinal progenitor formation in the uninjured retina in zebrafish [41]. The SB216763 drug, when used to stabilize β-catenin, caused an upregulation of *egr1* and enhanced retinal progenitor number. This increase of retinal progenitors was nullified by *egr1* knockdown. The *egr1* promoter had several TCF/LEF binding sites favoring β-catenin binding for transactivation. Negative regulation of Wnt signaling using the XAV939 drug caused a decline in retinal progenitor but enhanced *malat1* levels. However, this reduction in retinal progenitors because of XAV939 could be nullified by *egr1* overexpression. Thus, Egr1 could be an essential effector of Wnt signaling in stimulating proliferation during retina regeneration in zebrafish.

*egr1* mRNA is expressed in retinal progenitors, while *malat1* lncRNA is restricted to neighboring cells. The reduction of *egr1* levels upon *malat1* knockdown in the retina suggests that Delta-Notch signaling mediates their interplay, prompting us to explore its role in this regulation, and blocking one of the Delta-Notch signaling effectors, Her4.1, a transcriptional repressor, results in a reduction of retinal progenitors. We speculated if Her4.1 negatively regulated *malat1* expression. We confirmed that Her4.1 negatively affected *malat1* transcription. More importantly, in the *her4.1* knockdown retina, where proliferating cells are sparsely present, *malat1* expression was no more excluded from these cells.

Interfering with the Tgf-β signaling had a profound effect on zebrafish *malat1* levels. Zebrafish *malat1* promoter activity decreased with active Tgf-β signaling but increased with SB431542 treatment. Despite containing the pSmad3 transactivation domain, the 5GC element, the zebrafish *malat1* promoter appeared ineffective. Human *MALAT1* is known to be regulated via the antisense transcript *TALAM1*, whose presence dictates the stability and functionality of the *MALAT1* RNA [50]. Interestingly, the zebrafish *talam1* promoter had several TIE elements bound by pSmad3, thus contributing to its transcriptional repression. This inhibition of zebrafish *talam1* by Tgf-β signaling could be crucial in the zebrafish *malat1* dynamics. *egr1* levels also decrease in a manner similar to zebrafish *malat1*, on activating Tgf-β signaling. However, it is to be noted that despite a decline in *talam1*, mature *malat1*, and *egr1* in active Tgf-β signaling in zebrafish, it did not cause a decline in the retinal progenitor as expected with zebrafish *malat1* knockdown. This could be possible because of many other gene expression events during the early and late phases of retina regeneration, regulated during retina regeneration through Tgf-β signaling [47]. Presumably, the downregulation of *talam1*, *malat1*, and *egr1* in activated Tgf-β signaling in zebrafish played a balancing role in restricting the retinal progenitor number within an optimal range, as done by Delta-Notch signaling in regenerating zebrafish retina [39]. Together, these findings lead us to hypothesize that the differential regulation of *malat1* in mice and zebrafish through TGF-β signaling could also account for the lack of retinal regeneration in mice compared to zebrafish. Further experiments are warranted to completely understand the mechanistic differences in *malat1* regulation between zebrafish and mice retina.

Exploration of TGF-β signaling during mice retina regeneration demonstrated that mouse *Malat1* and *Talam1* levels are upregulated because of TGF-β1 protein delivery. The *Egr1* levels also get positively regulated in a TGF-β1 protein concentration-dependent manner in mice retina. However, being anti-proliferative, the TGF-β signaling in mammals did not cause retinal progenitor proliferation, unlike zebrafish [47, 63]. In this study, we demonstrated that TGF-β signaling inhibited *egr1* expression in zebrafish, a finding that contrasts with observations in mice, where TGF-β signaling positively regulates *Egr1*. In zebrafish, blocking TGF-β signaling led to upregulation of the pro-proliferative gene *egr1*, which did not increase retinal progenitors. Surprisingly, co-overexpression of TGF-β1 and *egr1* caused a decline in progenitor cell numbers. Various reports show that TBX2 protein interacts with Egr1 and sequesters it away from its target sites [53, 64]. We saw that TGF-β signaling induced *tbx2a* expression, a pro-proliferative factor, while *egr1* downregulated it. The elevated levels of TGF-β1 and *egr1* significantly upregulated *tbx2a* levels, suggesting the co-sequestration of Tbx2a and Egr1, nullifying the pro-proliferative effect of each other, although this interaction remains to be further explored. In mice, TGF-β signaling positively regulated *Egr1*, possibly via *Malat1*, but their simultaneous elevation could inhibit *Egr1*’s pro-proliferative function due to *Tbx2* interplay. This finding highlights how the same pathway can produce opposing effects on proliferation depending on species and context. While promising, further investigation is needed to confirm these preliminary results, and modulating Egr1 during active TGF-β signaling may offer a potential therapeutic strategy to enhance mammalian retinal regeneration.

## Supplementary Material

ugaf024_Supplemental_File

## Data Availability

The data underlying this article are available in the article and in its online supplementary material.
