# Supplementary material for: Regulation of the lncRNA malat1/Egr1 axis by Wnt, Notch, and TGF-β signaling: a key mechanism in retina regeneration
Source: NAR Mol Med. 2025 Jul 21;2(3):ugaf024. doi: 10.1093/narmme/ugaf024 (PMC12430008; doi:10.1093/narmme/ugaf024)
Supplement: ugaf024_Supplemental_File [file ugaf024_Supplemental_File.pdf]

Supplementary Figures

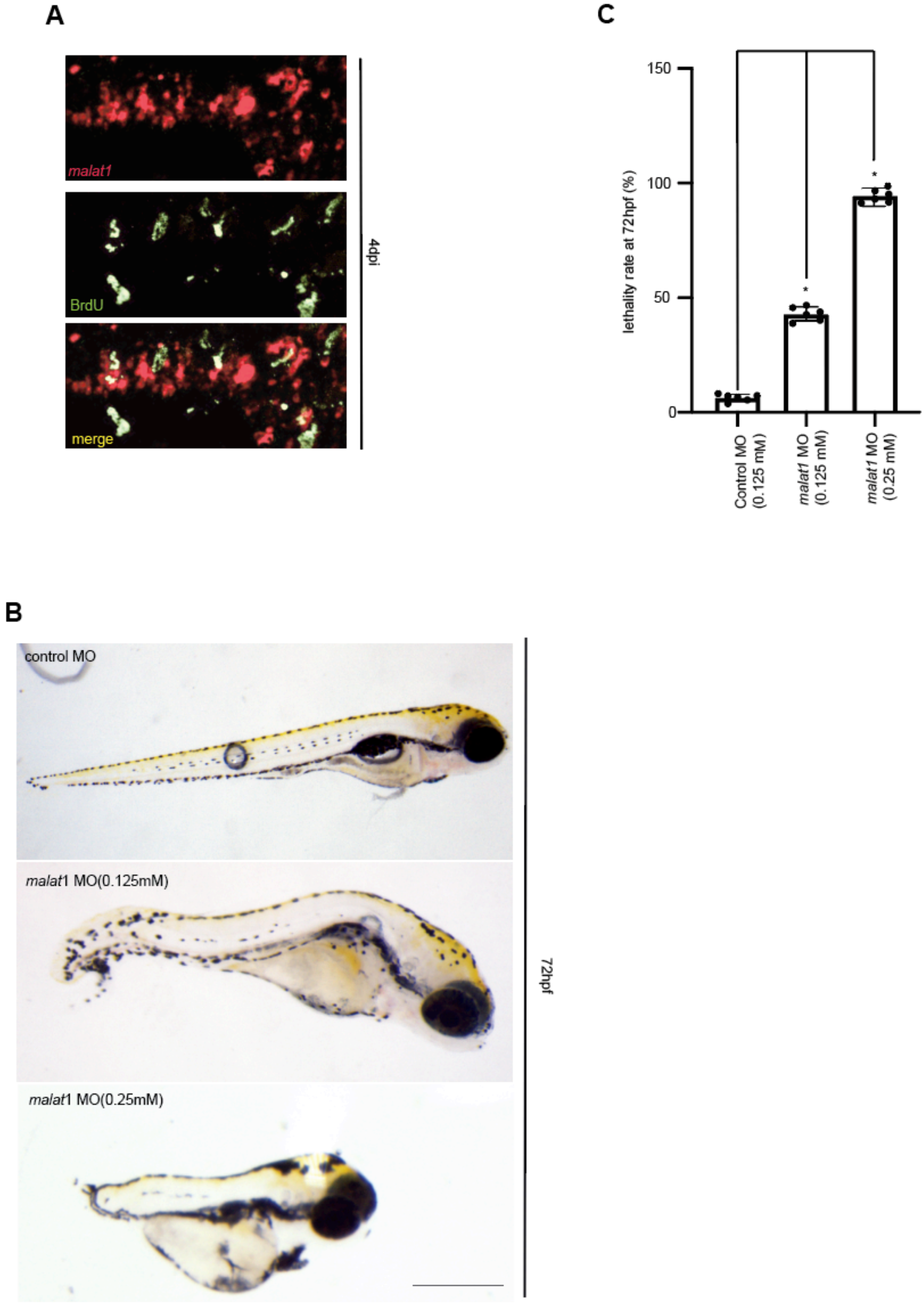

Figure S1: ***malat1*** **is expressed in cells around proliferating cells, and is essential for zebrafish embryonic development and survival.**
(A) Confocal images after fluorescence *in-situ* hybridization of *malat1* at 4dpi, on retinal cross-section immunostained with BrdU, to mark proliferating cells at 4dpi. **(B)** Bright-field

images of zebrafish embryos at 72 hours post-fertilization (hpf) demonstrating morphological defects following *malat1* knockdown using varying concentrations of morpholino.
(C) Graph depicting the lethality rates of zebrafish embryos at 72 hpf upon injection with different concentrations of zebrafish *malat1* morpholino. The data reveal a dose-dependent increase in lethality with increasing morpholino concentrations. Error bars represent standard deviation. *$p < 0.005$; $n > 100$ embryos analyzed per morpholino group. Each injection was repeated >5 times. Morpholino was injected into the yolk of one-cell stage embryos. Scale bar in **(A)**:10 µm and **(B)**:1mm.

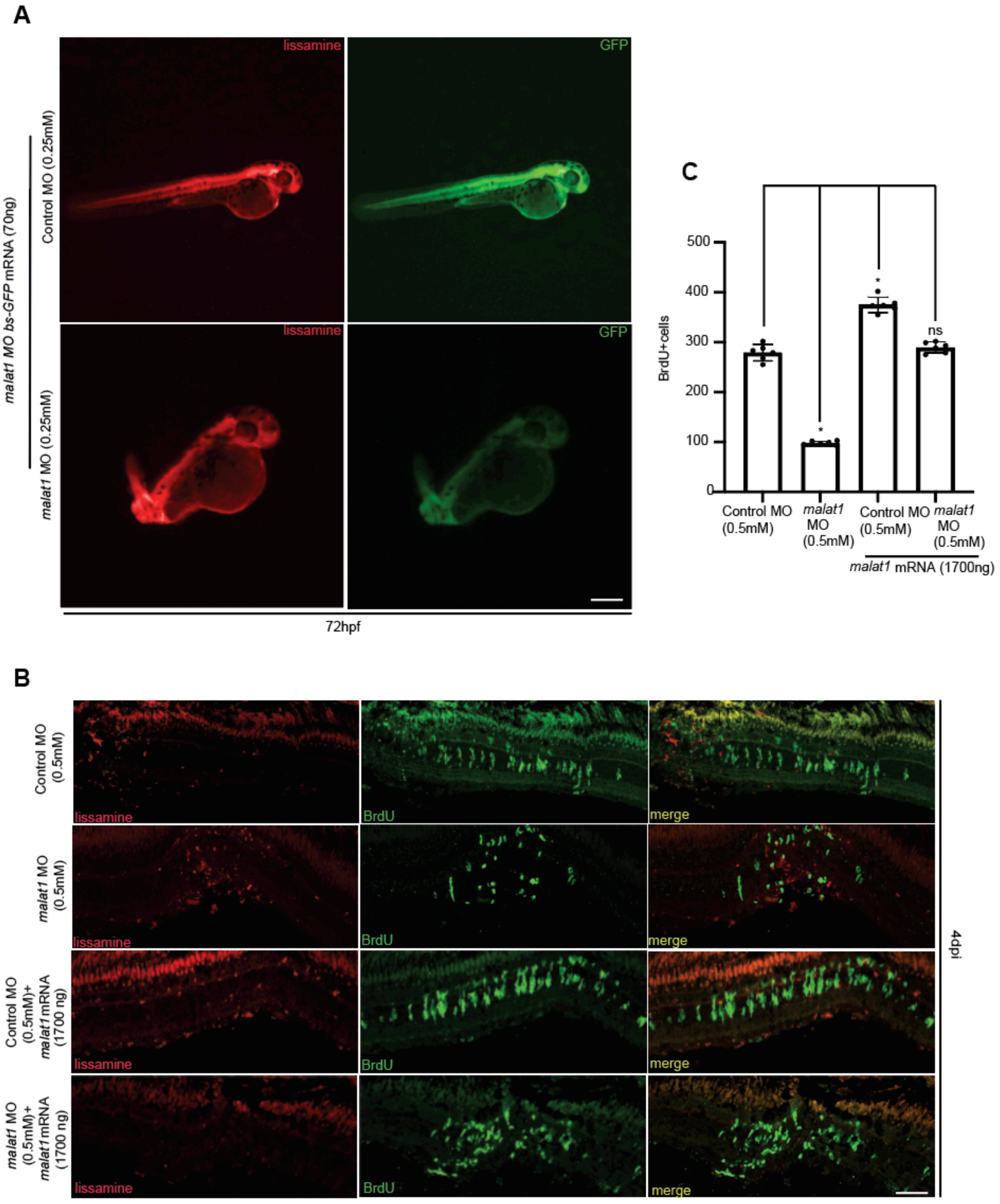

Figure S2: *malat1* overexpression had a positive impact on MGPC proliferation, and rescued the effect of *malat1* MO.

(A) The fluorescence image of a 36 hpf zebrafish embryo demonstrates reduced GFP expression upon injection with zebrafish *malat1* MO and mRNA containing the zebrafish *malat1* MO binding site (bs) upstream of *gfp*, compared to embryos injected with control MO and mRNA containing the zebrafish *malat1* MO binding site (bs) upstream of *gfp*. **(B)** Confocal images of retinal cross-sections at 4 dpi showed increased BrdU+ cells upon *malat1* RNA transfection, highlighting its pro-proliferative role. *malat1* knockdown reduces BrdU+ cells, an effect rescued by zebrafish *malat1* overexpression. Data is presented as mean ±

standard deviation (SD), *p < 0.05; n = 6 biological replicates. Quantification is shown in **(C)**. Scale bar: 1mm **(A)** and 10 µm **(B).** Injury model: Mechanical needle poke

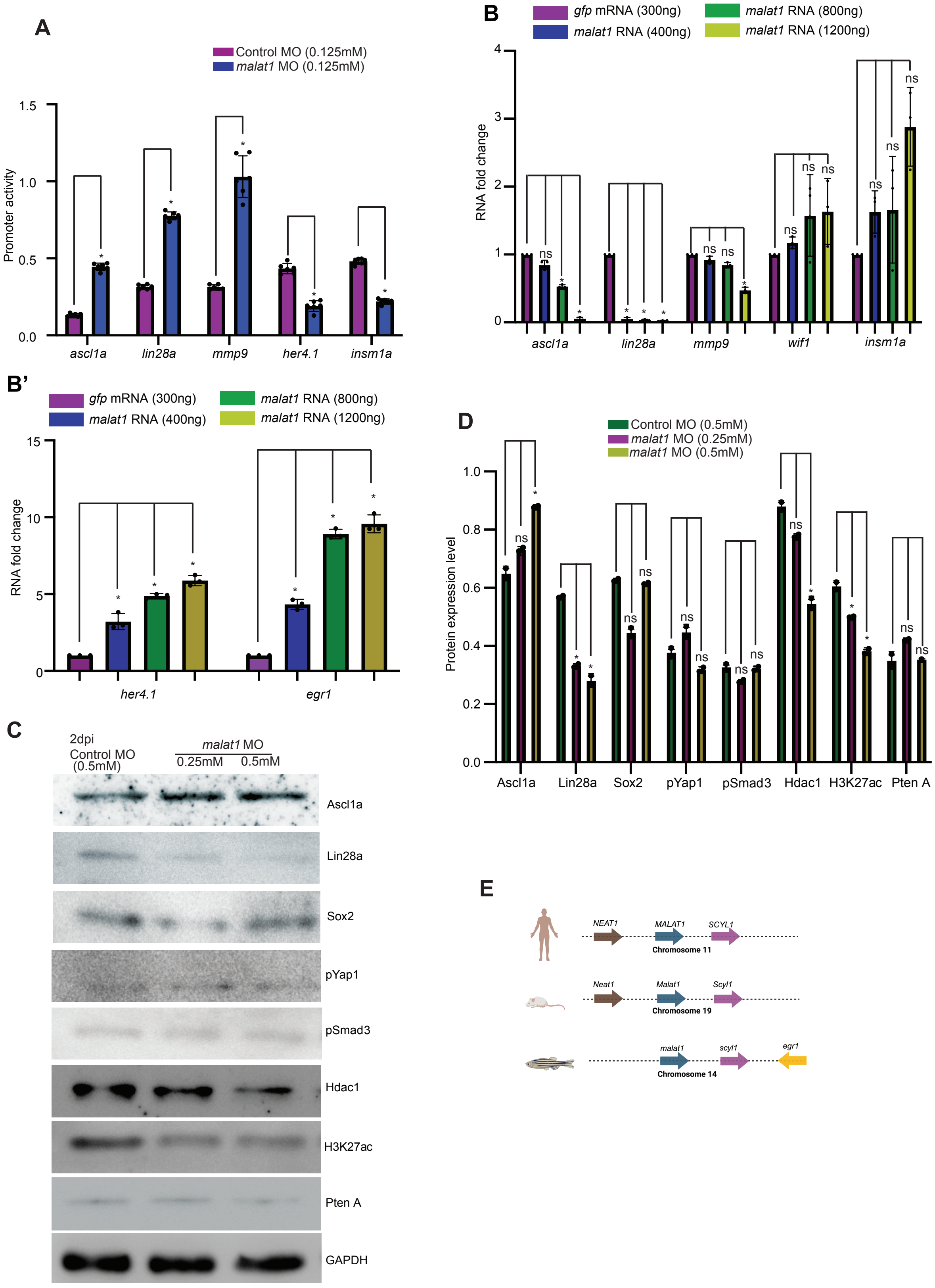

Figure S3. ***malat1*** **knockdown alters the expression of regeneration-associated genes and affects chromatin status.**

(A) Luciferase assay in zebrafish embryos (24hpf) showing the dysregulation in *ascl1a, lin28a, mmp9, her4.1,* and *insm1a* promoter activity on zebrafish *malat1* knockdown. Data is presented as mean ± standard deviation (SD), *p < 0.04; n = 3 biological replicates. **(B)** and **(B')** qPCR analysis of RAGs (*ascl1a, lin28a, mmp9, wif1, insm1a, her4.1* and *egr1*) on zebrafish *malat1* overexpression at 2dpi in zebrafish retina. . Data is presented as mean ± standard deviation (SD), *p < 0.05; n = 3 biological replicates. ns indicate non-significant. Injury model: Mechanical needle poke **(C)** Western blot analysis shows changes in the expression of regeneration-associated proteins, epigenetic modifiers, signaling molecules, and epigenetic marks on chromatin following *malat1* knockdown at 2 dpi. These findings suggest that *malat1* impacts a wide range of proteins and modulates the chromatin status of cells to mediate its effects. **(D)** Quantification of western blot band intensities, performed using ImageJ software **(E)** A schematic depicting the arrangement of the *malat1* gene and its neighboring genes on linear DNA in humans, mice, and zebrafish.

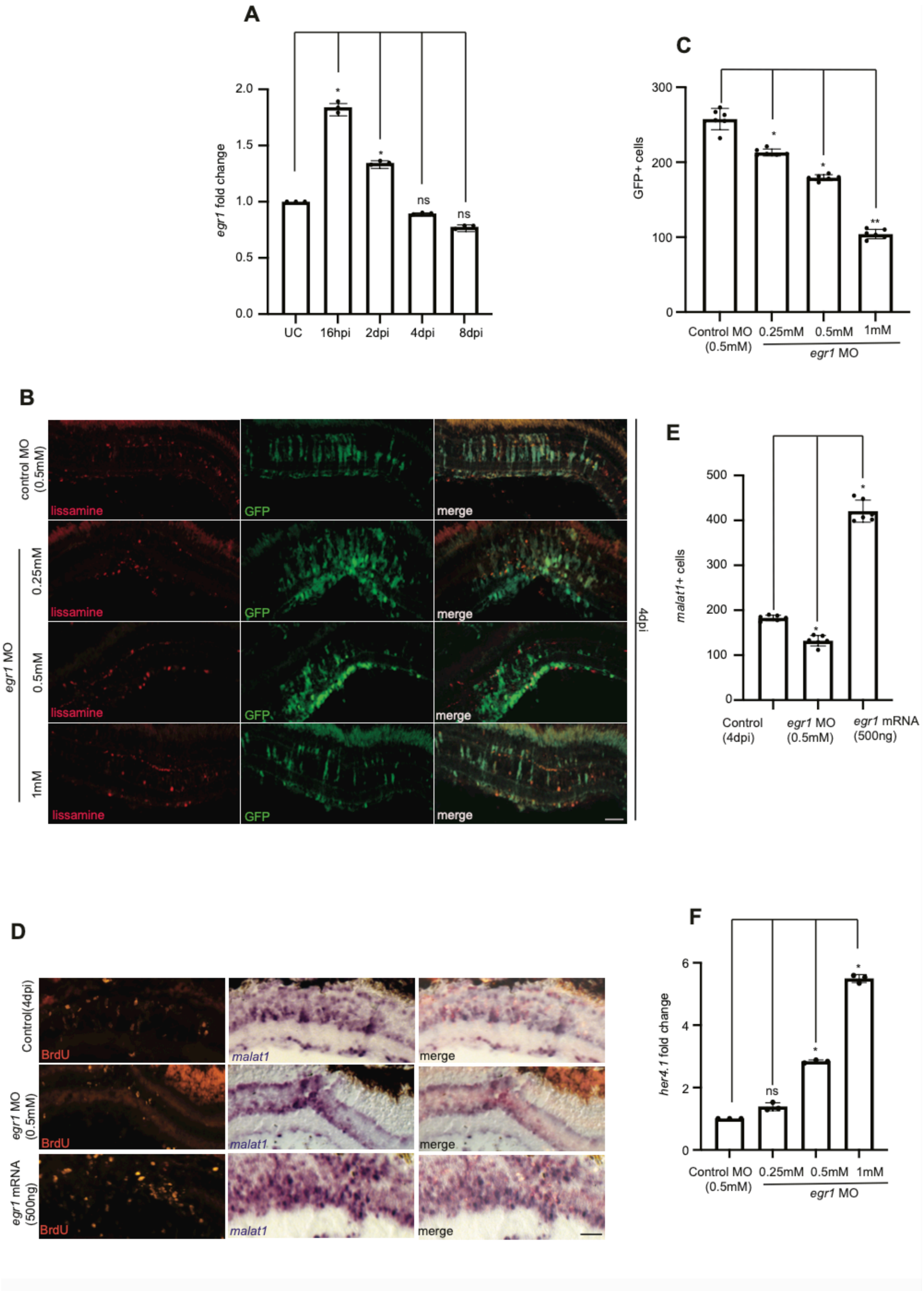

A
egr1 fold change
2.0
1.5
1.0
0.5
0.0
UC
16hpi
2dpi
4dpi
8dpi
ns
C
GFP+ cells
300
200
100
0
Control MO (0.5mM)
0.25mM
0.5mM
1mM
egr1 MO
B
control MO (0.5mM)
egr1 MO
0.25mM
0.5mM
1mM
lissamine
GFP
merge
4dpi
E
malat1+ cells
500
400
300
200
100
0
Control (4dpi)
egr1 MO (0.5mM)
egr1 mRNA (500ng)
D
Control(4dpi)
egr1 MO (0.5mM)
egr1 mRNA (500ng)
BrdU
malat1
merge
F
her4.1 fold change
6
4
2
0
Control MO (0.5mM)
0.25mM
0.5mM
1mM
egr1 MO

Figure S4: *egr1* exerts a pro-proliferative effect and form a positive feedback loop with *malat1* during zebrafish retina regeneration.
(A) qPCR analysis of *egr1* expression levels at various time points post-retinal injury, showing early induction of *egr1* after injury. Data is presented as mean ± standard deviation (SD), *$p < 0.05$; n = 3 biological replicates. **(B)** Confocal images of retinal cross-sections from the *tuba1a1016:gfp* transgenic line at 4 dpi, showing a decline in GFP positive proliferating cells upon *egr1* knockdown in a concentration-dependent manner, quantified in **(C).** Data is presented as mean ± standard deviation (SD), *$p < 0.0005$; n = 6 biological replicates. **(D)** Bright-field images of zebrafish *malat1 in-situ* hybridization following *egr1* knockdown and overexpression respectively, indicating that *egr1* positively regulates *malat1*, suggesting a positive feedback loop, quantified in **(E)**. Data is presented as mean ± standard deviation (SD), *$p < 0.005$; n = 6 biological replicates. **(F)** qPCR analysis of *her4.1* levels on *egr1* knockdown at 2dpi, in zebrafish retina. Data is presented as mean ± standard deviation (SD), *$p < 0.05$; n = 3 biological replicates. ns indicates non-significant. Scale bars represent 10 µm **(B, D)**. Injury model: Mechanical needle poke

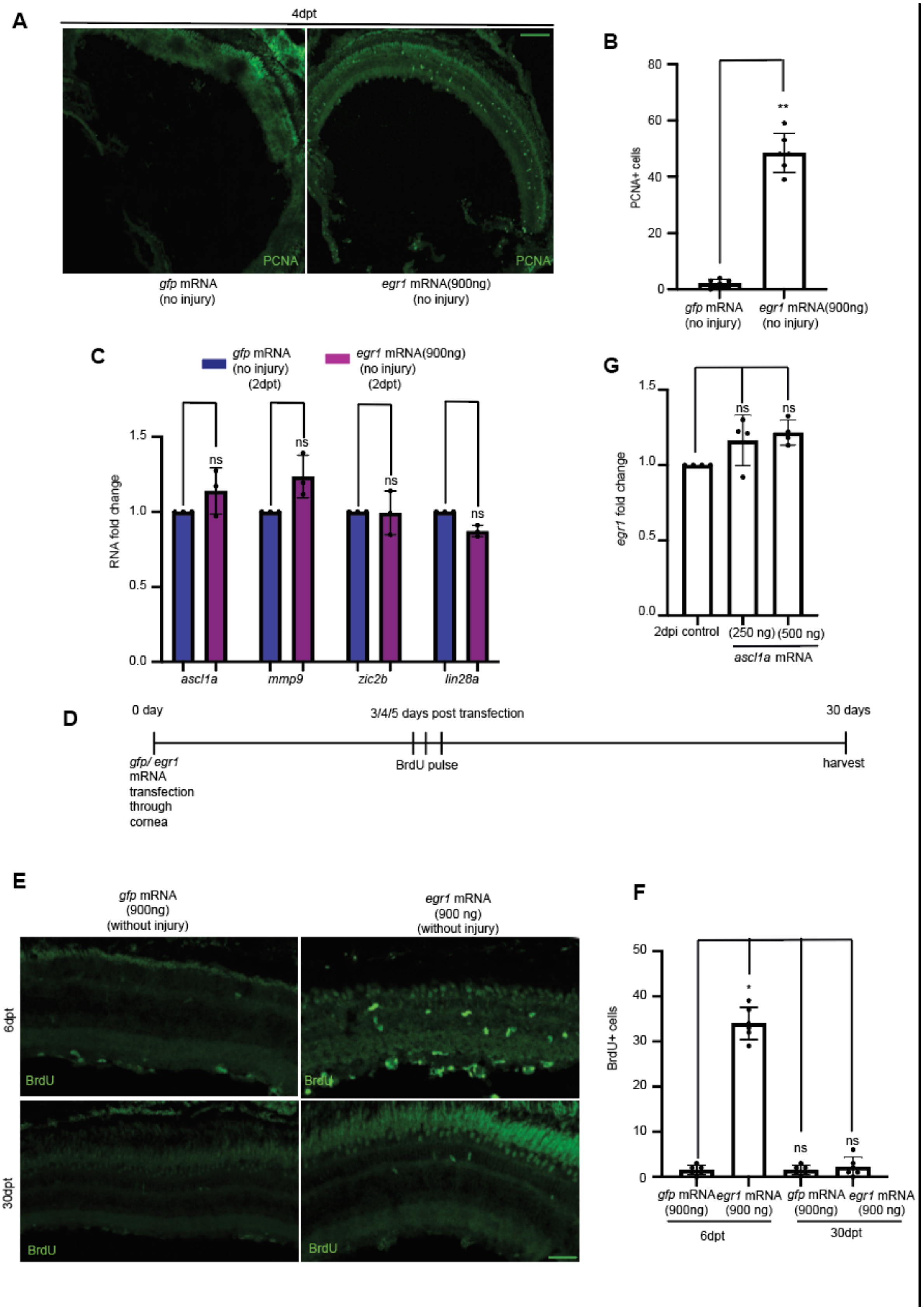

Fig S5: Egr1 can induce MGPC proliferation but requires sufficient induction of RAGs for effective regeneration to happen.

(A) Confocal images of zebrafish retinal cross-sections showing PCNA immunostaining in retinas where *egr1* was overexpressed without injury, indicating MGPC proliferation occurs even without injury, if *egr1* is overexpressed. Quantification is shown in **(B)**. Data is presented as mean ± standard deviation, $*p < 0.003$; n = 6 biological replicates. **(C)** qPCR analysis of regeneration-associated genes (RAGs) at 2 days post-transfection (dpt) with *egr1* overexpression in uninjured zebrafish retinae. No significant induction of RAGs was observed (ns indicates non-significant). Data is presented as mean ± standard deviation, $*p < 0.05$; n = 3 biological replicates. **(D)** Schematic representation of the experimental regime for evaluating the viability of proliferated MGPCs (cells labeled with BrdU at 3/4/5 dpt) after *egr1* overexpression without injury. **(E)** Confocal images of retinal sections show that while *egr1* overexpression induced MGPC proliferation at 6 dpt, these proliferated cells were not viable by 30 days post-transfection (30dpt), as no BrdU+ cells were observed at 30 dpt. **(F)** Quantification of MGPC proliferation and viability at 6 dpt and 30 dpt respectively, on *egr1* overexpression without injury. **(G)** qPCR analysis of *egr1* mRNA with overexpression of *ascl1a* mRNA, in 2dpi retina. Data is presented as mean ± standard deviation, $*p < 0.005$; n = 6 biological replicates. Scale bars represent 10 µm (**A, E**). ns indicates non-significant. Injury model: Mechanical needle poke.

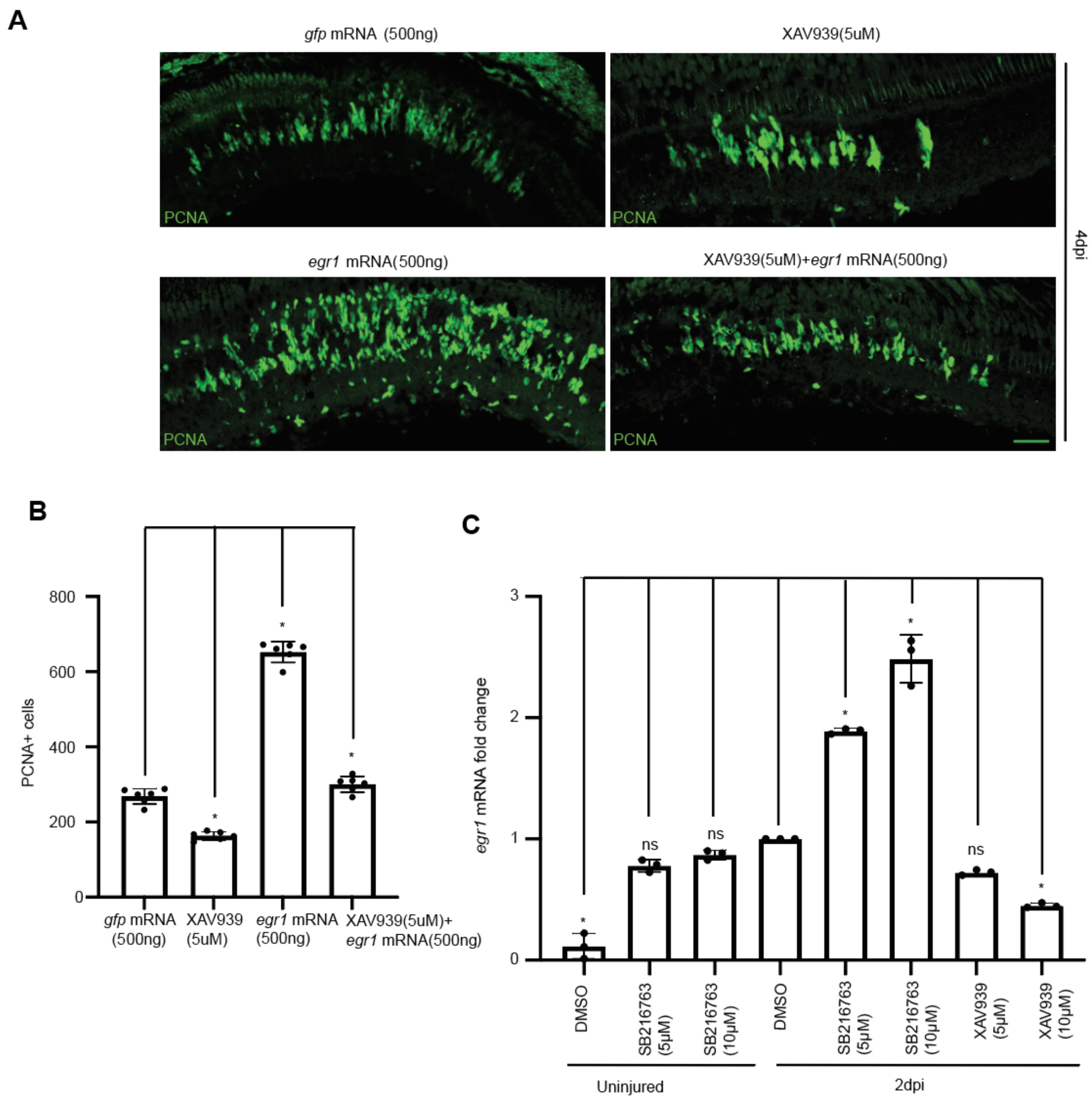

Figure S6: Wnt signaling regulates *egr1* during retina regeneration.
(A) Confocal images illustrating that the effect of Wnt signaling inhibition (using XAV939) on reducing proliferation is rescued by overexpressing *egr1*, quantified in **(B)**. Data is presented as mean ± standard deviation, *p < 0.003; n = 6 biological replicates. Scale bars represent 10 µm. **(C)** qPCR analysis shows that *egr1* gets induced by stabilizing β-catenin (by SB216763 administration), with or without injury. Conversely, *egr1* levels decline upon Wnt signaling inhibition (XAV939 administration), demonstrating a critical role for Wnt signaling in modulating *egr1* expression. Data is presented as mean ± standard deviation, *p< 0.05; n=3 biological replicates. ns indicates non-significant. Injury model: Mechanical needle poke

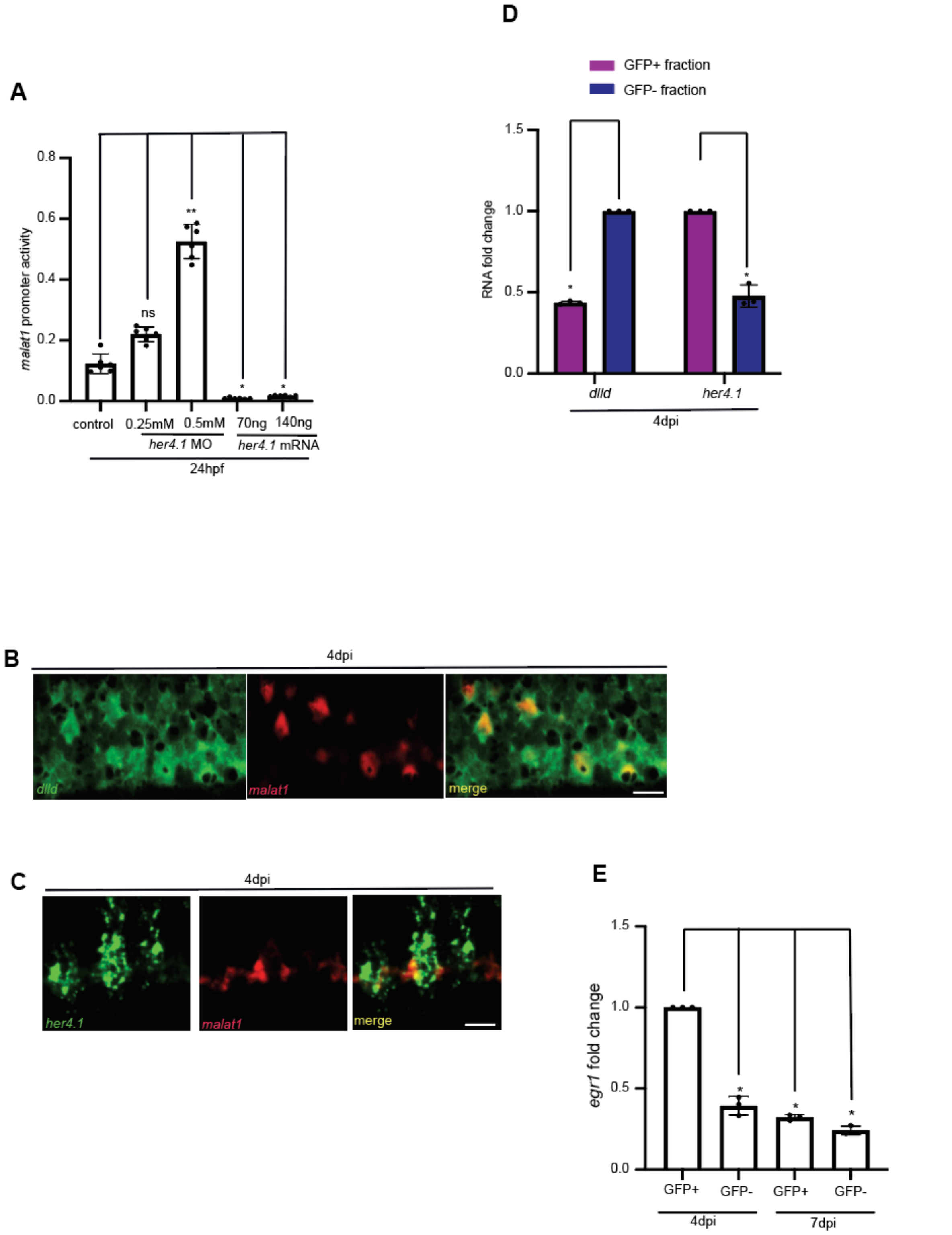

Figure S7: Notch signaling and Wnt signaling regulate *malat1* expression. Neighboring cells express *malat1* and *dlld*, while proliferating cells express *her4.1*

(A) Luciferase assay in zebrafish embryos (24hpf) supports that *her4.1* knockdown upregulates *malat1* expression, and *her4.1* overexpression suppresses *malat1* expression in zebrafish retina. **(B)** Double Fluorescence *in-situ* hybridization (FISH) shows *malat1* expressing cells have more *dlld* expression and **(C)** *malat1* expressing cells and *her4.1* expressing cells lie adjacent to each other. **(D)** qPCR analysis in sorted cells, from 4dpi

injured *tuba1016:gfp* transgenic retina, where GFP positive fraction contains proliferating cells, and GFP negative fraction contains non-proliferating cells, show *her4.1* enrichment in GFP positive (actively proliferating) and *dlld* enrichment in GFP negative (neighboring) fraction. Data is shown as mean values ± SD, *$p < 0.05$; n = 3 biological replicates. **(E)** qPCR analysis from the sorted cells reveal enrichment of *egr1* transcript in GFP+ fraction (actively proliferating cells). Data is shown as mean values ± SD, *$p < 0.05$; n = 3 biological replicates. ns indicates non-significant. Injury model: Mechanical needle poke

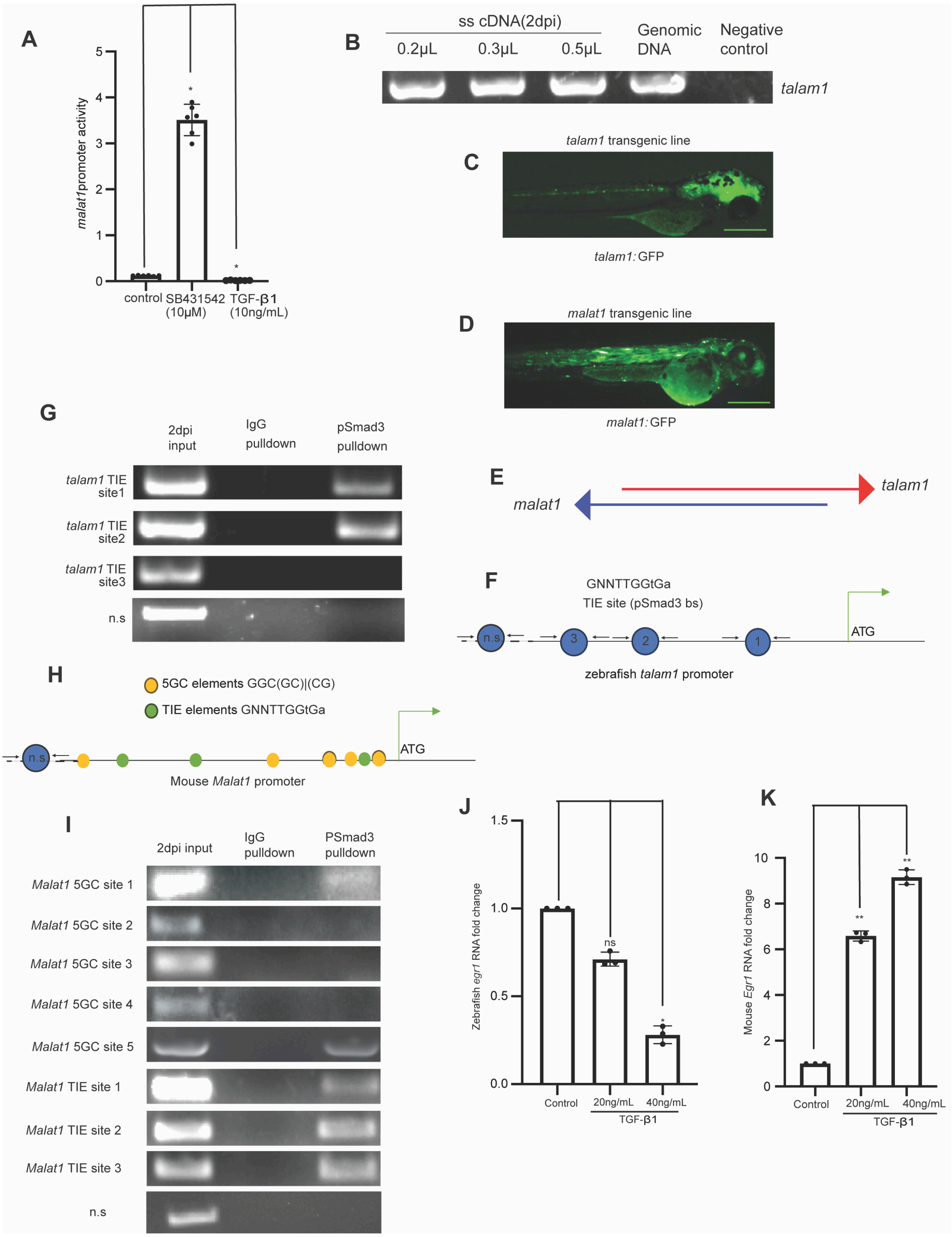

Figure S8: Differential regulation of *malat1* by TGF-β signaling in zebrafish and mice is exerted through direct binding of pSmad3 on regulatory elements of *talam1* promoter. TGF-β signaling regulates *egr1* expression in both zebrafish and mice like its regulation of *malat1*.

(A) Luciferase assay in zebrafish embryos (24hpf) supports that SB431542 (TGF-β inhibitor) and TGF-β1 overexpression upregulates and downregulates zebrafish *malat1* expression respectively in injured retina. Data is shown as mean values ± SD, $*p < 0.003$; n = 6 biological

replicates. **(B)** ssRT-PCR showing the presence of *talam1* transcript in zebrafish embryos, with genomic DNA as a positive control. **(C)** Fluorescent image of a 36 hpf *talam1* zebrafish reporter line showing GFP expression along the CNS, indicating *talam1* promoter activity in embryos. **(D)** transgenic *malat1* zebrafish reporter line embryo, showing similar expression of GFP along CNS, albeit stronger than *talam1* reporter line **(E)** Orientation of *malat1* and *talam1* genes. **(F)** Diagrammatic representation of TIE elements on the zebrafish *talam1* promoter. **(G)** PCR analysis after pSmad3 ChIP in 2 dpi zebrafish retinae shows pSmad3 occupancy on TIE elements of the zebrafish *talam1* promoter. **(H)** Diagram showing 5GC and TIE elements on the mouse *Malat1* promoter. **(I)** PCR after PSmad3 ChIP in mouse retinae shows PSmad3 occupancy on 5GC and TIE elements of the mouse *Malat1* promoter. **(J,K)** The qPCR analysis of *egr1* mRNA in zebrafish **(J)**, and *Egr1* mRNA in mice **(K)**, retina with different concentrations of TGF-β1 protein treated conditions. Scale bar: 1mm **(C)** and **(D).** n.s indicates non-specific site in ChIP PCR. Injury model: Mechanical needle poke.

Supplementary Table

**Primer name**	**Sequence (5'--- 3')**
malat1_RT_fwd	AAACCCATCCACTCGCTCTG
malat1_RT_rev	CTTATCTCCAGGTAGCGGCG
egr1_RT_fwd	ATTTCCATCTCAGCTTCCTCACCAG
egr1_RT_rev	GAAAGCGCAGACTGCATGTCG
Mouse malat1_RT_fwd	AGCTTTTGAGGGCTGACTGC
Mouse malat1_RT_rev	CGACTGGAGCACGAGGACACTGATTGTGGTAGGTCATCTGTTC
Mouse talam1_RT_fwd	GCACTAAAGACCACGGAACT
Mouse talam1_RT_ rev	CGACTGGAGCACGAGGACACTGAGCCTTTAGTCTCTTCCAGATT
Mouse egr1_RT_fwd	GAAGCCCTTCCAGTGTCGAATC
Mouse egr1_RT_rev	AAAAGGTCGCTGTCATGTCTGAAAG
Malat1_full length_fwd_BamHI_1_fwd	ATGCTAGCGGATCCGGGGTGAGGCGCTATGGAAG
Malat1_full length_rev_EcoRI_2_rev	ATGCTAGCGAATTCTTTACAGGGAAGTCAAACTGACTC
Malat1_full length_fwd_EcoRI_3_fwd	ATGCTAGGAATTCTTTAAAAGCTTCAAGGAAAG
Malat1_full length_rev_StuI_4_rev	ATGCTAGCAGGCCTTTCAAATTAATTTTTGCATATAAATACGTAACG

Malat1_in-situ_EcoRI_fwd	ATGCTAGGAATTCGGTGAGGCGCTATGGAAGG
Malat1_in-situ_XhoI_rev	ATGCTAGCTCGAGCCTTGCTGTACTGAAATGGTCGT
actin-RT-F	GCAGAAGGAGATCACATCCCTGGC
actin-RT-R	CATTGCCGTCACCTTCACCGTTC
talam1_rev_with linker	CGACTGGAGCACGAGGACACTGAGATCACGTCACCTGAATGCAAGTG
talam1_fwd	GGAGTTGAACCCCGTCCTG
Linker rev	CGACTGGAGCACGAGGACACTGA
Talam1_Promoter_pTal_XhoI_Fwd	ATGCTAGCCTCGAGATATATGAAGTCAGAATTATTAGCCCCCTTTAG
Talam1_Promoter_pTal_SalI_rev	ATGCTAGCGCTGACGTCGTGACGCACAATGTTGTTG
Egr1_full length_BamHI_fwd	ATGCTAGCGGATCCACCATGGCTGCAGCCAAGACAG
Egr1_full length_XhoI_rev	ATGCTAGCTCGAGTCAGCAGATGTCGGCTGTCCGAG
mouse gapdh fwd	CTGCCACCCAGAAGACTGTG
mouse gapdh rev	CTGTTGCTGTAGCCGTATTCATTGTC
pcs2.her4.1_EcoRI_fwd	ATGCTAGCGAATTCACCATGACTCCTACAATCACTGGATCAATC
pcs2 her4.1 xho1 rev	ATGCTAGCCTCGAGCTACCAGGGTCTCCAGATGTGAC
egr1 promoter tcf lef bs fwd 1	ATTTGTTGAGCGGTGTGATGAC
egr1 promoter tcf lef bs rev 1	CGATTTGTGTTGGGTCAAGATTAATG
egr1 promoter tcf lef bs fwd 2	GTTTGTAGCAATAGGTTCCCTCAAAC
egr1 promoter tcf lef bs rev 2	GTGTAGCAACATTCCCAAATACTTATGAC
egr1 promoter tcf lef bs fwd 3	GTCATAAGTATTTGGGAATGTTGCTACAC
egr1 promoter tcf lef bs rev 3	CTTGTAGCAATCTAATTGTAGGAGAGGC
mmp9_RT_fwd	GGAGAAAACTTCTGGAGACTTG
mmp9_RT_fwd	CACTGAAGAGAAACGGTTTCC
Ascl1a_RT_fwd	ATCTCCCAAAACTACTCTAATGACATGAACTCTAT

Ascl1a_RT_rev	CAAGCGAGTGCTGATATTTTTAAGTTTCCTTTTAC
Lin28a_RT_fwd	TAACGTGCGGATGGGCTTCGGATTTCTGTC
Lin28a_RT_rev	ATTGGGTCCTCCACAGTTGAAGCATCGATC
Her4.1_RT_fwd	GCTGATATCCTGGAGATGACG
Her4.1_RT_rev	GACTGTGGGCTGGAGTGTGTT
Insm1a_RT_fwd	CCAAGAAAGCCAAAGCCATGCGGAAGC
Insm1a_RT_rev	TTATTGCTTTCCGCGCTCTGCTTGGGTTTG
Zic2b_RT_fwd	ATGTTACTGGACGCCGGCCACCA
Zic2b_RT_rev	TTAAACGTACCACTCGTTAAAATTG
Wif1_RT_fwd	CTTCAAAACATGCCAGAGAGCAAAG
Wif1_RT_rev	CACATAGTTGGTTTCAGACGGCTGACTG
Cdk4_RT_fwd	ACGCAAGCCTCTGTTCTGC
Cdk4_RT_rev	CGCTGAAGAAGGGATGATCCAG
Ccnd2_RT_fwd	AGAGTTTGCTGACCATCGAAGAGA
Ccnd2_RT_rev	GTTGCCACCATCCTCCGCAT
Cdkn1_RT_fwd	GATCCTACGTTCACTCGGTAATG
Cdkn1_RT_rev	GTTCTGGTGTTTTCGGGATGTTTTC
Malat promoter_5GC_site1_fwd	GAACACGCAAACTCCACAC
Malat promoter_5GC_site1_rev	GTTCGTCTTTTGGCCCTTCATAAG
Malat promoter_5GC_site2_fwd	CATTTGACCCAACCCTCTTC
Malat promoter_5GC_site2_rev	CTCCGAACAGAAACGACAAC
Talam1 promoter_TIE_site1_fwd	GGAAATTGATAAAGGGTTCGTTTCTCAGAAC
Talam1 promoter_TIE_site1_rev	TTTCCCCAGTGTCTGTAGTTTTTTTTTATAGAC
Talam1 promoter_TIE_site2_fwd	GCTCCAAATAAGGAGGATAGTGTATTTTTTCATATG

Talam1 promoter_TIE_site2_rev	ATCACGCCTATCTTACAGTCTACTACTG
Talam1 promoter_TIE_site3_fwd	TTTATATGACTTGAACCTGCTCCCGA
Talam1 promoter_TIE_site3_rev	TACGTTTTTATGCGCATTTTCAAAAATTATGTACATC
Mouse malat1 promoter_5GC_site1_fwd	TGTACCCGGGCTCGGAAAAG
Mouse malat1 promoter_5GC_site1_rev	ATTTTGTAACTCCCCGAGCGG
Mouse malat1 promoter_5GC_site2_fwd	CTTGGGGCTCCCATTTTTAATAGC
Mouse malat1 promoter_5GC_site2_rev	GACGCAGAGCCCGGAG
Mouse malat1 promoter_5GC_site3_fwd	CTGCTCCCACACCAGTCATTC
Mouse malat1 promoter_5GC_site3_rev	GAGCACCACCCGCACTG
Mouse malat1 promoter_5GC_site4_fwd	TGTCTACCCTGGGAAAGCCTCTG
Mouse malat1 promoter_5GC_site4_rev	GTTGATCCCGTAAACTGGAGGCTG
Mouse malat1 promoter_5GC_site5_fwd	GTTTCTCTGTGTATCTCTGGCTGTC
Mouse malat1 promoter_5GC_site5_rev	CTGGAGGCCAGGGAAATGAAAC
Mouse malat1 promoter_TIE_site1_fwd	GTCGCCGTGCCCTCA
Mouse malat1 promoter_TIE_site1_rev	CGAGTGGCTTCTGAGGTG
Mouse malat1 promoter_TIE_site2_fwd	GTGGGCATTTGGGCCCTAG
Mouse malat1 promoter_TIE_site2_rev	CTGCAGATAAGGGAAACTAGGCAAG
Mouse malat1 promoter_TIE_site3_fwd	CTCATCCTCTCTTCCTGCACATTAG
Mouse malat1 promoter_TIE_site3_rev	CTGAGCTGCATGCCTAACTGTC
Delta d_RT_fwd	AAATGGAGGAAGTTGCACTGATC
Delta d_RT_rev	AAGATCGAGACACTGAGCATCATTC
malat1 mo bs-pegpn1_gfp upstream_fwd	CAGTCGACGGTAAACCATGCGGAAAAAAAGCAAAAGACCCTGGT GGCCGCGGGCCCGGGATC
malat1 mo bs-pegpn1_gfp upstream_rev	GATCCCGGGCCCGCGGCCACCAGGGTCTTTTGCTTTTTTTCCGCAT GGTTTACCGTCGACTG
Tbx2a_RT_fwd	CAACCGAGTGATCATCAAGCACT

Tbx2a_RT_rev	CTTTCCCAGTGATGGGTTGCAG
Malat1 splice site_fwd_ClaI	ATGCTAGCATCGATGTCACCTGAATGCAAGTGCAGC
Malat1 splice site_rev_StuI	ATGCTAGCAGGCCTGAGGAGCACTTGCTCTACATCA
Malat1 promoter_fwd_XhoI	ATGCTAGCCTCGAGCAACTGACCCAGCCTGTGAGGCG
Malat1 promoter_fwd_EcoRI_pel	ATGCTAGCGAATTCGAGGCAGTGATGCGCTCGCAG
Malat1 promoter_rev_SalI_pTal	ATGCTAGCGTCGACGAGGCAGTGATGCGCTCGCAG
Dlld T3_rev_insitu	AATTAACCCTCACTAAAGAAGATCGAGACACTGAGCATCATTC

Table S1: The list of primers and sequences used in this study
